# ChemEngine: harvesting 3D chemical structures of supplementary data from PDF files

**DOI:** 10.1186/s13321-016-0175-x

**Published:** 2016-12-29

**Authors:** Muthukumarasamy Karthikeyan, Renu Vyas

**Affiliations:** 1 Chemical Engineering and Process Development (CEPD), CSIR-National Chemical Laboratory, Pashan Road, Pune, Maharastra 411008 India; 2 MIT School of Bioengineering Sciences and Research, ADT (Art, Design and Technology) University, Loni Kalbhor, Pune, Maharashtra 412201 India

**Keywords:** Chemoinformatics, Supplementary data, Organic reaction modeling, Density functional theory, Text mining, Data mining

## Abstract

Digital access to chemical journals resulted in a vast array of molecular information that is now available in the supplementary material files in PDF format. However, extracting this molecular information, generally from a PDF document format is a daunting task. Here we present an approach to harvest 3D molecular data from the supporting information of scientific research articles that are normally available from publisher’s resources. In order to demonstrate the feasibility of extracting truly computable molecules from PDF file formats in a fast and efficient manner, we have developed a Java based application, namely ChemEngine. This program recognizes textual patterns from the supplementary data and generates standard molecular structure data (bond matrix, atomic coordinates) that can be subjected to a multitude of computational processes automatically. The methodology has been demonstrated via several case studies on different formats of coordinates data stored in supplementary information files, wherein ChemEngine selectively harvested the atomic coordinates and interpreted them as molecules with high accuracy. The reusability of extracted molecular coordinate data was demonstrated by computing Single Point Energies that were in close agreement with the original computed data provided with the articles. It is envisaged that the methodology will enable large scale conversion of molecular information from supplementary files available in the PDF format into a collection of ready- to- compute molecular data to create an automated workflow for advanced computational processes. Software along with source codes and instructions available at https://sourceforge.net/projects/chemengine/files/?source=navbar.

**Electronic supplementary material:**

The online version of this article (doi:10.1186/s13321-016-0175-x) contains supplementary material, which is available to authorized users.

**Graphical abstract:**
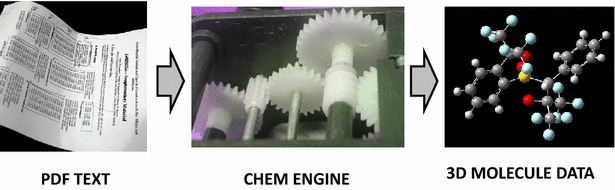
.

## Background

 Harvesting chemical data from the web is a challenging task requiring several convoluted steps. When chemical structures are stored in truly computable format with atoms and bond matrices (vector format-Cartesian co-ordinates), they can be processed electronically for computational and informatics purposes. However while transforming/storing the files in PDF (Printable/Portable Document/Data Format) that are usually used for the convenience of printing and reading, the valuable and re-usable molecular data is totally lost and buried in scientific literature as documents and seldom used for further computational studies. In earlier days, the hand-drawn molecules in ORTEP diagram formats were published while discussing the 3D conformation of molecules in the research articles. Generation of 3D structures from these molecular images in raster format was extremely difficult. Recently, some efforts have been made to transform computer generated and hand-drawn chemical images from journal articles and patent documents into truly computable molecules for inventory and database applications. Other similar endeavors include transforming either the textual chemical names (common, systematic, corporate identifiers for example CAS Registry number) or the computer generated names into corresponding molecular structures with moderate success. Although the name to chemical structure conversion programs are now routinely being used for harvesting chemical data from documents yet they have been insufficient in generating the accurate and truly computable and re-usable molecular data. The supporting information related to computational methods based research articles, describing the transition states of organic reactions is now available from journal publishers’ websites containing description of computations performed with tables of results, molecular images in 3D conformations along with 3D molecular co-ordinates in a PDF format. This combined data in a single file complicates the harvesting process and development of pattern recognition techniques for selectively excluding the non-atomic co-ordinate information from the pool of large collection of textual data presented as supporting material. Since there are no defined rules and guidelines for submitting molecular data in a supporting document associated with research publications, the authors are free to choose their favorite methods of representing molecular data such as chemical structures and corresponding atomic co-ordinates in the supplementary data file. This freedom of choosing data formats necessitates the development of several pattern recognition templates in the form of regular expressions to handle diverse formats (co-ordinates separated by space, comma, tab etc.) and maintain the order in which the XYZ co-ordinates and atom information is presented by the authors. This study therefore highlights the need for development of standards required for submitting the supporting materials with molecular data in a consistent, truly computable and re-usable format to journals publishing computational research. A specific set of guidelines defined by the publishers to submit molecular data even in a PDF format, would accelerate the automatic processing and recognition of chemical data for further computational studies related to reaction modeling [1–3], drug-discovery [4–7] and molecular inventory management [8, 9]. Several standard molecular representations in ASCII format which are easily readable by molecular modeling and chemoinformatics software packages are available. Supporting materials are deposited in PDF format for the convenience of storage, easy manageability and electronic dissemination. The commercial software packages applied for computational chemistry applications employ their own legacy file formats for handling molecular data, the technical details of which are not usually published. From the researchers’ point of view, the published data in re-usable formats would save efforts and time to understand the molecular data better and use it for practicing to carry out further advanced studies in different problem solving environments that require 3D conformation of molecules. Exchange of chemical data between multiple softwares without loss of information is a critical requirement in computational chemistry and chemoinformatics applications. Thus there is a need for the development of tools that can bridge the gap in molecular data translation automatically and accurately from PDF format to truly computable, re-usable format without manual intervention.

In this context, it is pertinent to mention the efforts by Rzepa and Peter Murray-Rust for developing tools to parse chemically relevant thesis and other published articles for harvesting analytical data [10, 11]. Special emphasis was laid on the use of Information Technology (IT) techniques for free re-distribution of electronic chemical data, for instance, storing actual supplementary information in structured XML/CML documents for universal applicability and dissemination of the valuable experimental/computed data thus advancing “data led science” as is the case in biology. The blue obelisk informal group initiative [12], encourages the use of open source data, open standards, shared algorithms and tools for performing chemoinformatics tasks. It has led to the development of valuable tools such as JChemPaint [13], CDK [14] and chemical information systems [15]. Similar efforts have been made by the Cambridge Crystallographic Data Center (CCDC) group that provides easily downloadable crystal structures of organic molecules that are pliant with a number of software solutions for drug discovery [16]. In a recent article, the importance of curation of large chemogenomics data set for building better predictive model for life sciences has been emphasized [17]. During the preparation of this manuscript, a timely research article by Rzepa’s group on granularity model for extracting molecular information appeared [18] that stresses on the need for periodic and automatic curation of data from supplementary information in research articles. The present work is geared towards partial fulfillment of this need for “futuristic research data management”.

Conventionally, chemical names (common, systematic), Chemical Abstract Registry numbers are extracted from the web-pages and transformed into corresponding molecular structures using name-to-structure conversion tools [19], name to structure relational database look-up methods [20], large scale key-value pair list [21], distributed relational database search [22] etc. We have previously employed distributed systems to harvest chemical data using Google API (ChemXtreme) from the web pages [23]. Transforming the raster images into vector graphics followed by identification of relevant pixel information associated with atoms and bonds of a molecule is a cumbersome job [24]. Tools have also been developed to harvest molecular data from images using web camera, scanned images wherein the raster graphics data was transformed into vector graphics to eventually retrieve the atoms and bonds information for the generation of truly computable and re-usable chemical structures such as ChemRobot [25], OSRA [26], ChemReader [27], CLiDE [28], but only limited success has been achieved. A foolproof method with complete reproducibility of computable molecules from images is still a distant dream as the existing methodologies and tools do not provide accurate molecule data after processing. Therefore it is essential to develop efficient tools that can extract molecules from rich sources such as supplementary information files deposited at the journal site. Although spectral, molecular and analytical data have been harvested in the past but extracting molecules directly from author supplied atomic coordinates provided in supplementary materials as PDF format is not known. Accordingly, in the present work, we have developed an application, ChemEngine that reads all the files stored in the PDF format to extract molecular coordinates and generate computable molecular structures. To demonstrate the efficiency of the program, supporting material data files of three different molecular representations in terms of delimiters in the co-ordinate data were selected and the data was successfully parsed using ChemEngine to extract molecular data. It is to be noted here that the first two files from ACS publications did not require permission for data harvesting, while in the third case (RSC Advances), an article published under the CC-BY license was selected. It is also observed that the bulk processing of articles or supporting materials from publishers’ site automatically is usually prohibited due to copyright and article access policy.

**Fig. 1:**
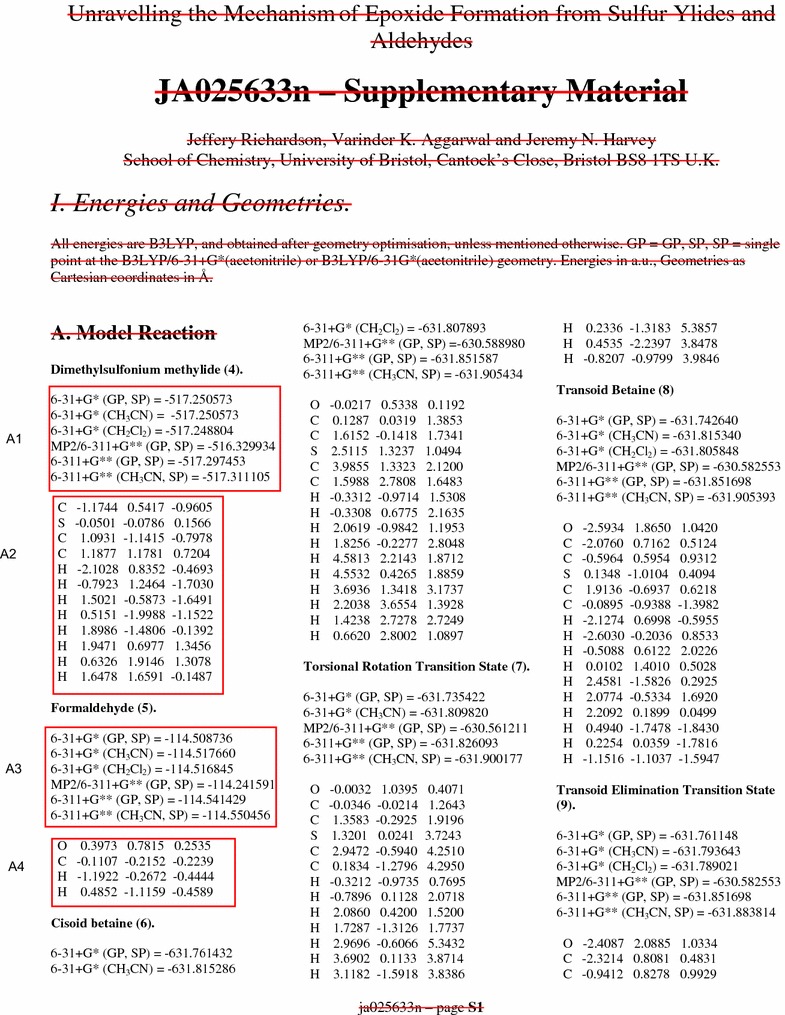
Supplementary data of a journal article (case study I) depicting the computed molecular data format, the contents in the *highlighted text* are required for the re-computation of data. A1, A2, B1, B2 refer to text patterns in the specific document. The *crossed out text in red color* is ignored while generating the coordinate file by ChemEngine version 1.0

Generally every software program dealing with computational chemistry, provides an export format for the computed data either as a plain text or delimited text that can be analyzed, visualized, plotted via common tools like Microsoft excel or other molecular viewers that accept molecules as plain text in simple.xyz formats. However, supporting materials of molecular data files also include brief description of molecules, computed data, plots, page numbers, document information, manuscript bibliographic details etc. as a single document in PDF format that makes harvesting the molecular data extremely difficult as these have to be selectively excluded while parsing the file. In the Fig. 1, only the enclosed text in the rectangular box is correctly recognized using patterns by ChemEngine, the rest of the unstructured text is ignored. Given an input file in PDF format, the program yields three different files in GJF format, text file containing computed bond matrix and all molecules in SDF format. The contents of the non molecular data file can also be utilized by further subjecting it to standard text mining methodologies [29, 30] for retrieving molecule names or other information such as list of basis sets employed in the specific computational work.

## Implementation

**Fig. 2:**
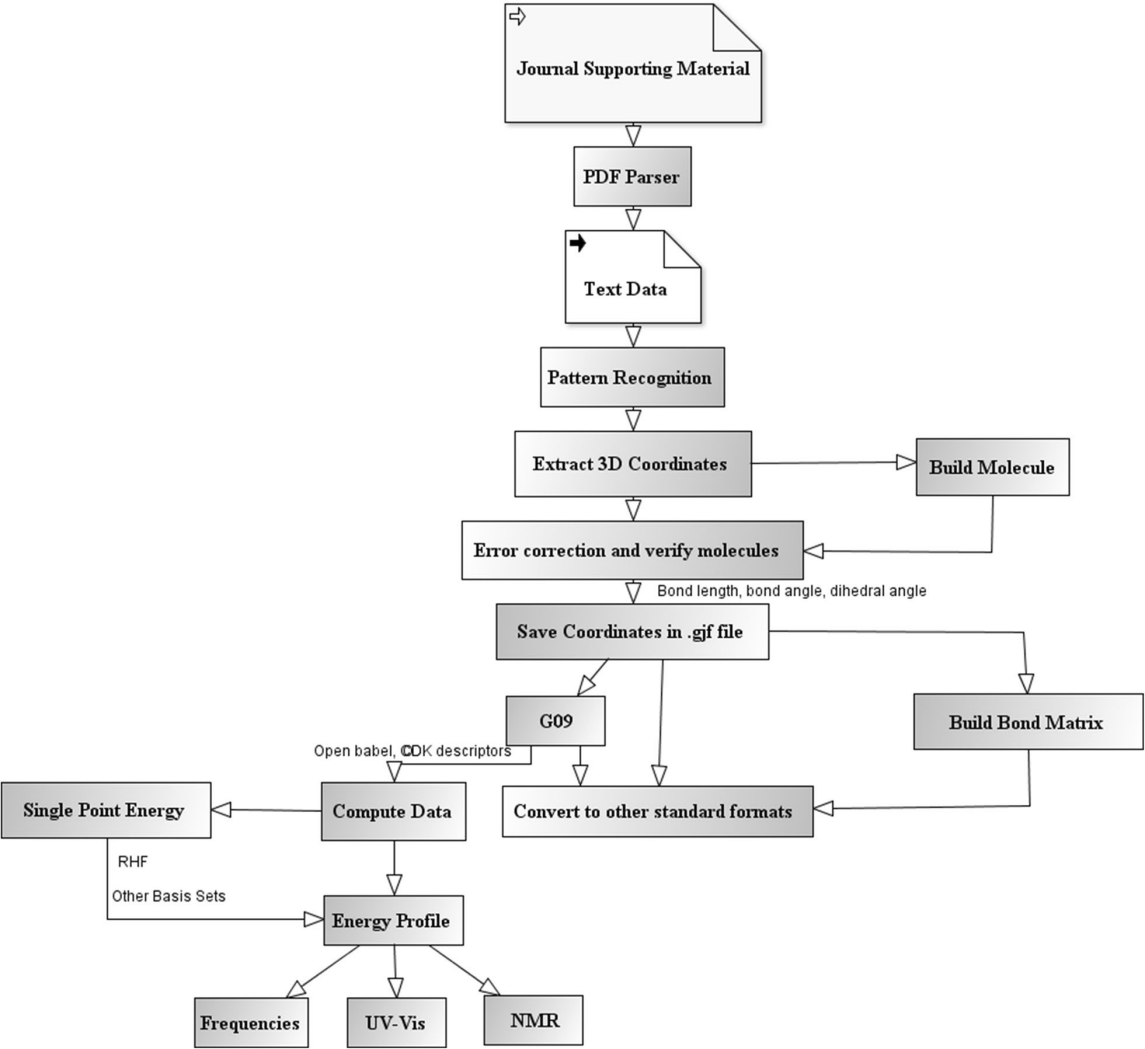
Computational steps workflow for extracting re-computable molecular structures from PDF articles as implemented in ChemEngine

The ChemEngine application was deployed on a computer with Intel Xeon(R) CPU E5-2603 (2 Processors), 16 GB RAM, 1 TB hard disk running 64 bit operating system on Windows Server 2008 Enterprise. The computational steps employed in the Java based ChemEngine application are highlighted in the flowchart (Fig. 2).

ChemEngine is updated with default option to accept PDF file containing molecular coordinates. Internally the program recognizes the textual and non-textual data and using a default pattern recognition method to separate the 3D coordinates from the non-molecular text for the identification of atomic co-ordinates and atom information. The pseudo code with generic regular expression for harvesting atomic coordinate data from the input file is shown below.

### Pseudo code

(Co-ordinate Text).matches (“Regular Expression Pattern with Delimiter Definition”);

For Example: Delimiter: Comma

String_Data.matches(“^[A-Za-z0-9]{1,2}\\,[0]{0,1}[\\,]{0,1}-{0,1}.{1,2}[0-9]{1,10}\\,-{0,1}.{1,2}[0-9]{1,10}.{1,}”)

Delimiter: Space

String_Data.matches(“^[A-Za-z0-9]{1,2}\\s+[0]{0,1}[\\s +]{0,1}-{0,1}.{1,2}[0-9]{0,10}\\s+-{0,1}.{1,2}[0-9]{0,10}.{1,}”)

**Fig. 3:**
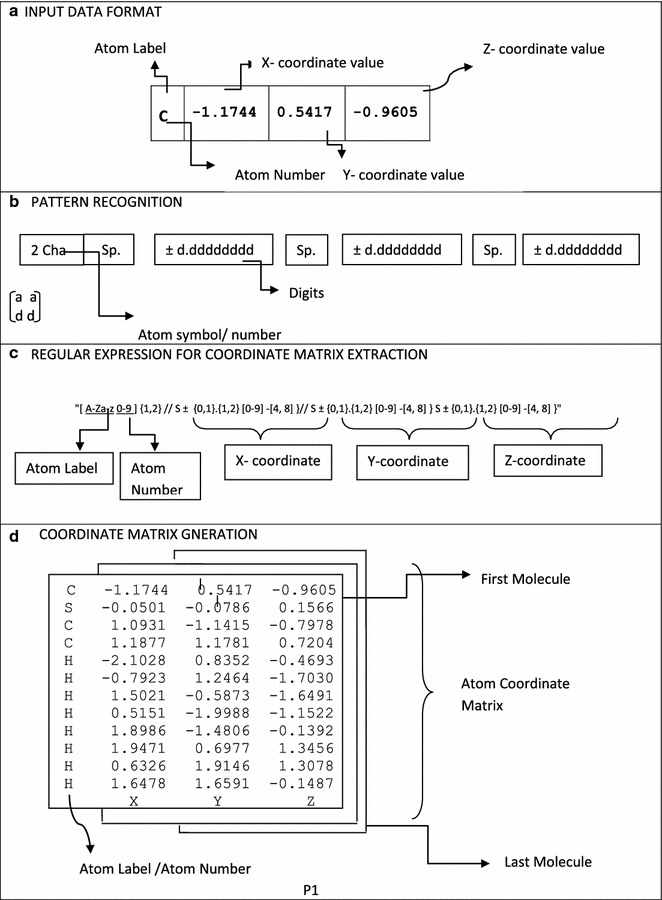
Detailed illustration of key steps in regular expression based pattern recognition for the generation of coordinate matrix data

The details of the derivation of the regular expression patterns from the coordinate data format can be viewed in Fig. 3. All the X, Y, Z coordinates were encoded by a general pattern sequence consisting of 2 characters, followed by a space, an addition or subtraction symbol, a number, decimal and eight digits succeeding the decimal.

Once the coordinate file is created, the bond matrix is computed to generate the atomic connectivity information for reconstructing the original molecules reported in the supplementary material of the research article. Important parameters such as bond angles, bond lengths and dihedral angles are verified and checked for consistency in the recreated molecule and then saved in the original file format, for instance gjf [31]. The coordinate data and bond matrix information is used to create molecules in standard interoperability formats such as .sdf or .mol as ready to compute molecules for the convenience of the user. This process avoids unnecessary generation of molecular data and laborious recomputation of already published work. The molecules can be subjected to further simulations such as descriptor calculation, energy profile, docking etc. The java based ChemEngine program is made available freely for non commercial purposes through the sourceforge site for evaluation and testing.

**Fig. 4:**
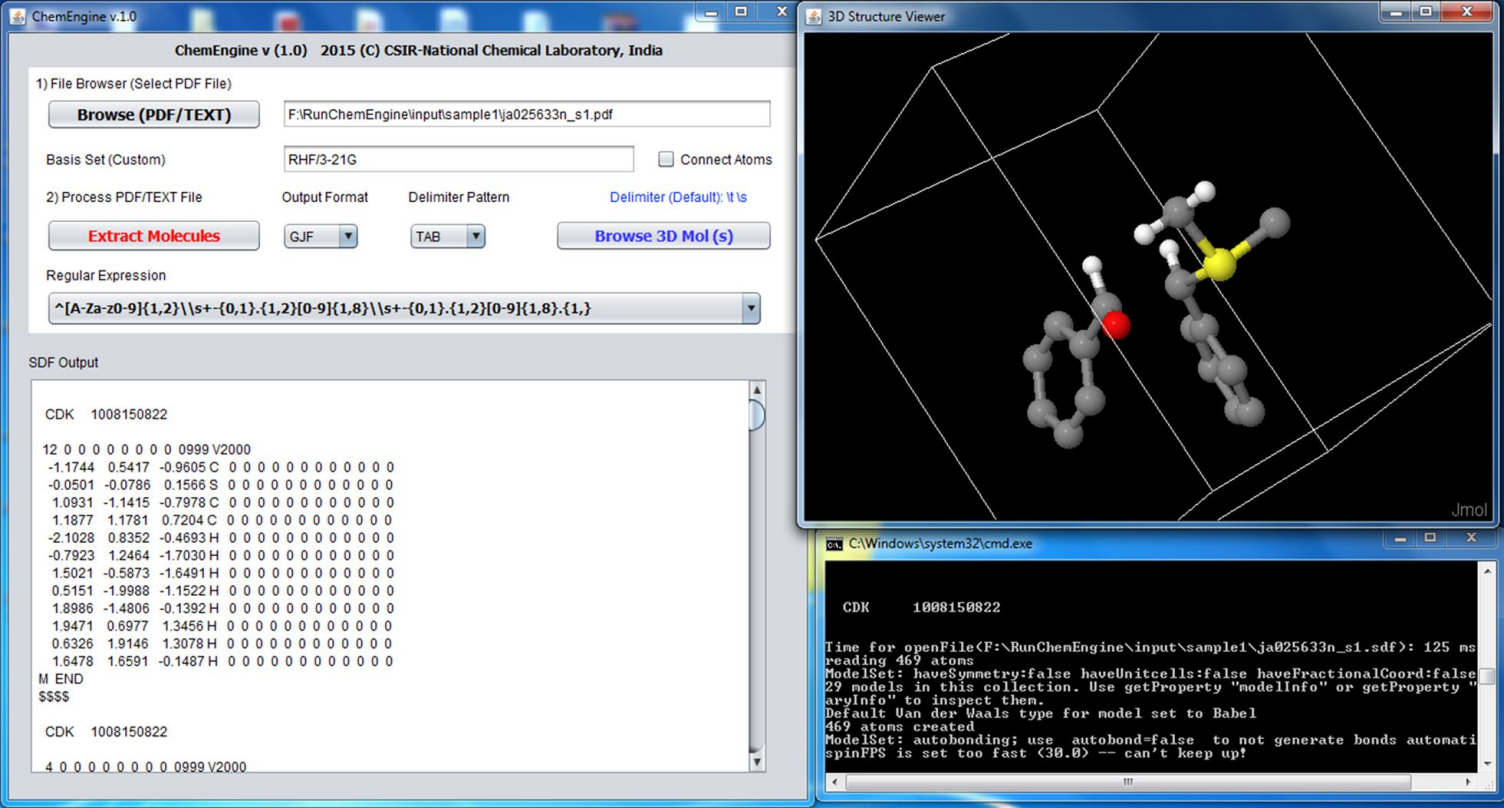
ChemEngine GUI for extracting molecular information from the supplementary file selected by the user. The extracted molecules can be dynamically viewed in a 3D viewer

A user friendly GUI has been developed for easy processing of PDF files, navigation and 3D structure generation (Fig. 4). The ChemEngine main screen displays the input file browser, the output display text area and a text box for specify regular expression. The GUI is enabled with a 3D molecule viewer (JMOL) for browsing the molecules in a pop up window. The program can also be used in the command line mode for automatic generation of SDF for the given PDF files. The user can either browse a locally stored PDF file or upload a text file for generating coordinates. Depending upon how the coordinates data is deposited in a supplementary file, there is a provision to specify the delimiter such as tab, comma and space if required. The user can select the desired regular expression to extract the coordinate data. Further, in future a customized regular expression can be incorporated into the system based on a particular journal standards of accepting coordinate data in a PDF file. On clicking the *connect atom* button in the browser window, the connection table for a group of coordinates representing a molecule is created and displayed in the output text area. The molecules thus recreated are stored as GJF and SDF format for future computational use and other database oriented inventory applications.

## Results and discussion

**Fig. 5:**
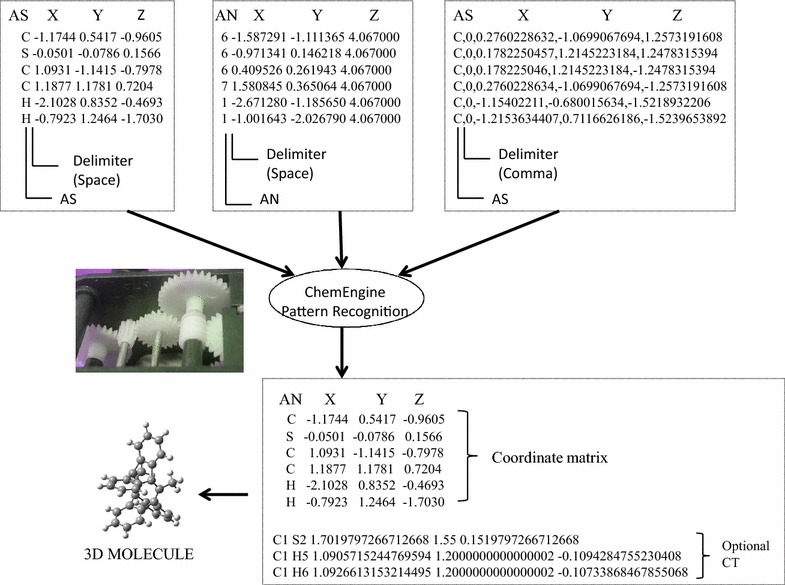
A schematic diagram highlighting the challenges posed by the diverse coordinate formats present in the supplementary table of journal articles selected for this study. ChemEngine identifies the text patterns and processes this information to yield a common generic format of coordinate matrix. Further the bond matrix algorithm implemented in the program generates a bond matrix for the creation of a connection table to generate the ready to compute 3D molecular structure. *AN* atomic number, *AS* atomic symbol, *CT* connection table

In general the major problem in processing molecular data stored in PDF files arises due to the non-standard representation of coordinates such as inconsistency in the number of digits appearing after the decimal, interchange of atom type with atomic number in the first column and improper alignment of x, y, z coordinate values. Three case studies have been chosen each dealing with a different representation of coordinate data format in the supplementary information. In the first case, ChemEngine could directly handle the given pdf file and extract the coordinate information. The process in the second case was not straight forward (due to error in PDF to text converter) so the PDF file was first saved as a. txt file externally and then processed to get the desired molecular data. The third case was even more challenging as the molecular coordinates were published in a comma delimiter form (Fig. 5).

### Case study 1

The supporting material file was related to reaction modeling research paper describing the mechanistic investigation of epoxide formation from sulfur ylides and aldehydes [32]. The work provided guidelines on stereo-selective synthesis of epoxide ring systems. The computational data included optimized geometries, calculated single point energies, rotational profiles and potential energy surface (PES) generation using standard B3LYP based DFT method. The PDF file was processed to directly extract a.txt file from which patterns were discerned to generate the bond matrix data. For a complete list of coordinate data of molecules generated by ChemEngine please refer to Additional file 1. This file can be considered as a standard template for submitting coordinate data of molecules for fast processing of PDF files in future.

An important constraint for generating ready to compute molecules was the non-availability of bond order information in the published coordinates data. Accordingly functionality has been built in ChemEngine for creating a bond matrix i.e. inter-atomic connectivities of a given cluster of atoms, to facilitate its recognition by the program as a molecule. This enables construction of the connection tables of molecules to assist the direct conversion of a PDF file to SDF on the fly. The method accurately retained the original conformations of all the optimized molecules when the extracted atomic coordinates were supplied back to the original program (Additional file 2).

**Fig. 6:**
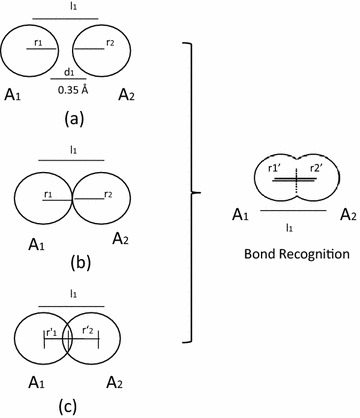
A schematic depicting the bond recognition logic implemented in ChemEngine v 1.0. **a**–**c** represent three scenarios between two interacting atoms *A1* and *A2*, wherein a bond was considered to be present

Understanding the atomic (electronic) movements and distances is of paramount importance in transition state modeling studies of organic reactions. Typically the cut-off distance for the presence of a bond is computed as the sum of the covalent radii of the two atoms, but researchers generally prefer to conduct a computationally less intensive QM calculation and determine based on Wiberg bond order as implemented in the QMDFF code [33]. We took into account the interatomic distances of all the elements in periodic table to annotate the bond order between two atoms. The logic implemented in ChemEngine for creating a bond matrix between two atoms A_1_ and A_2_ in a molecule is schematically represented in Fig. 6. The cut off distance between two vicinal atoms involved in a covalent bond formation was calculated as the sum of atomic radii + a scaling factor of 0.35 Å, any distance higher than this was considered as a non bonding interaction by the program. Likewise all interatomic distance of other atoms were computed to generate bond matrix of a molecule.

**Fig. 7:**
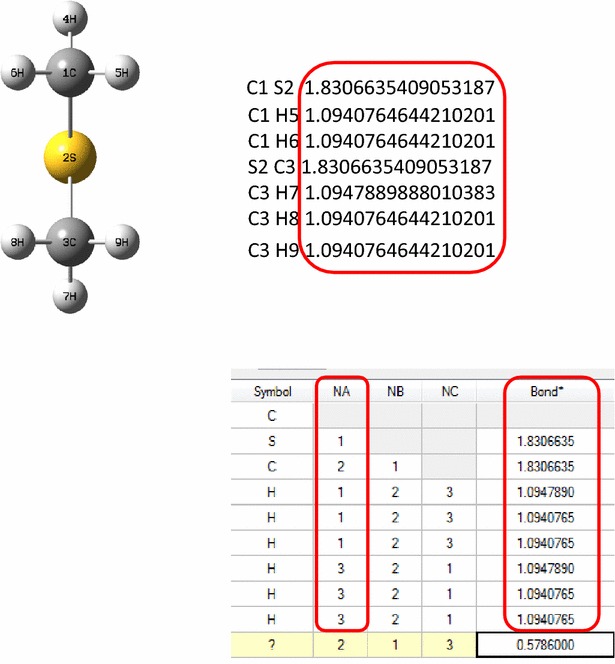
Interatomic bond distances reproduced using ChemEngine by harvesting the 3D coordinate structural data from the pdf file (Mol ID 29, Dimethyl sulfide). The bond characteristics were identical with those generated by the original program (GaussView)

To validate our method, the bond matrix for atoms of all the molecules (n = 29) deposited in the supplementary information of the research article was computed and compared with the ones generated by the original software (Gaussian). The values were identical in both the cases. Bond matrix conformation of a representative molecule from this set is shown in Fig. 7 (Bond matrix of few more molecules is shown in Additional file 3). The coordinate data and the computed connectivity information could be used to generate molecules in the SDF and MOL formats.

### Case study 2

The work pertains to a well cited paper wherein computational studies were performed on a range of alkenes to gain insights into the mechanistic processes involved in the thiol ene reactions [34] typically classified under click chemistry. In contrast with the previous case study, where the approach was straight forward and an open source pdf reader could be employed to convert pdf to text from the supporting information submitted in a pdf file, in the present case the pdf file was first saved in a plain text format externally and then submitted to ChemEngine for extracting the coordinates. The inadvertent errors in file conversion could be related to compatibility issues associated with various PDF maker programs available on the web.

ChemEngine program could successfully generate the Cartesian coordinates, bond matrix and non molecular data of all the reported molecules (Table 1). Due to the pagination problem in the original PDF document, only few structures partially failed (few atoms carry forward to next molecule) by the program. This pagination issue was later addressed by molecular block identifier—a simple subroutine with the help of which the program could correctly identify molecules reported in a document.

**Table 1:**
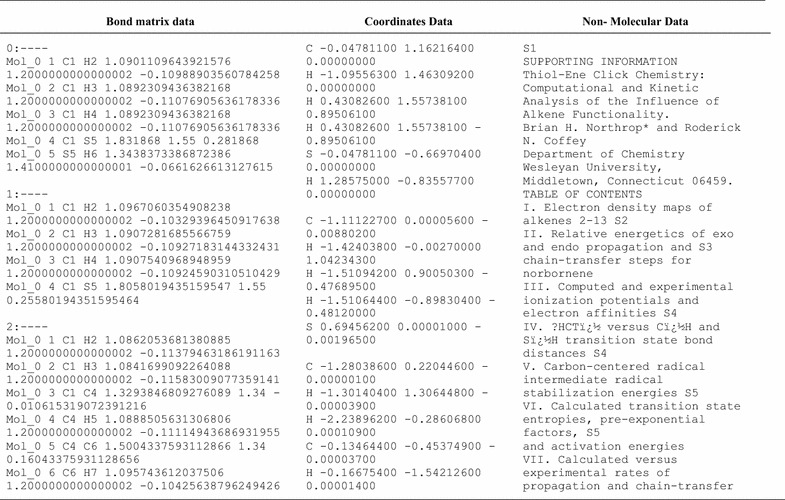
Sample data from three output files viz. bond matrix, coordinate data and non-molecular text generated by ChemEngine in the second case study related to thiol ene click chemistry. Data displayed here has been truncated for brevity (n = 115)

**Fig. 8:**
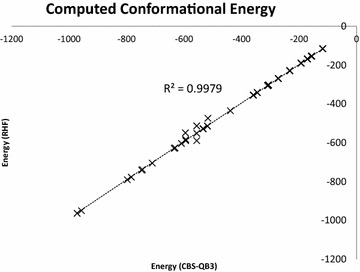
A comparative plot of single point energies of molecules extracted from coordinate data related to case study 2. The values are in agreement with the original computed data. *CBS* Complete Basis Set, *RHF* restricted Hartree–Fock

To establish the reusability of the molecular conformations extracted from ChemEngine, we computed their single point energies. RHF (restricted Hartree–Fock) method, a central starting point for multi electron systems was employed for quick computations. The resultant energy values were plotted against the original reported free energies derived from CBS-QB3 (Complete Basis Set), a computationally intensive composite method for yielding very accurate energies. Both the energies were in close agreement (R2 = 0.998) despite the choice of different methods (Fig. 8).

### Case study 3

The computational work reported involved Cope rearrangement transition states using the DFT method to compute electronic energies for various substituted allyl derivatives [35]. This example consisted of a PDF file wherein the coordinates data was submitted in a comma separated format in the supplementary file. The code implemented in ChemEngine was modified to parse any coordinates data interspersed with delimiters such as comma, tab or space in a PDF file. The results of all three case studies are summarized in Table 2 which prove the robustness and efficiency of the ChemEngine program in recognizing patterns and developing regular expression for the typical cases dealt (Additional files 4, 5).

**Table 2:** Details of the three case studies representing the diversity of coordinate molecular data in supplementary material handled by ChemEngine

Entry	Case study	N = molecules	Regular expression pattern	Format and delimiter
1	Epoxide formation from sulfur ylides and aldehydes	29	^[A-Za-z0-9]{1,2}\\s+-{0,1}.{1,2}[0-9]{1,8}\\s+-{0,1}.{1,2}[0-9]{1,8}.{1,}	PDF Space
2	Thiol ene click chemistry	115	^[A-Za-z0-9]{1,2}\\s+-{0,1}.{1,2}[0-9]{1,8}\\s+-{0,1}.{1,2}[0-9]{1,8}.{1,}	Text Space
3	Design of tetra(arenediyl)bis(allyl) derivatives for cope rearrangement transition states	55	^[A-Za-z0-9]{1,2}\\,[0]{0,1}[\\,]{0,1}-{0,1}.{1,2}[0-9]{1,10}\\,-{0,1}.{1,2}[0-9]{1,10}.{1,}	PDF Comma

### Case study 4

In order to increase the scope of this work to handle several hundred PDF files to harvest truly computable molecular data, that are buried in PDF files we have implemented a default option in ChemEngine to harvest atomic co-ordinate data mixed with images (spectral data, barcode images, experimental data, molecular description and other computed data) and successfully tested with several PDF files to regenerate molecular files without any errors [36].

In the present work we process the molecules and transform them into SDF format that is mostly compatible with commercial packages thus saving time and computational effort. The compute once and use many times approach will help the readers to access the original input files even after passage of time. It is pertinent to mention here that the biological sciences and bioinformatics community follow a standard representation of molecular coordinates in the PDB file format which is a database compliant format instead of a PDF format thus securing an easy access and exchange of information. Extracting coordinates of protein molecule from a PDF file, assuming an average protein size of over 2,00,000 atoms would have been indeed a truly challenging task. However with the aid of ChemEngine customized with additional atomic co-ordinate pattern recognition modules, now it is possible to harvest any molecular data from PDF format. With the advent of 3D structure repositories and several free academic sites, data storage is no longer a major issue, the ready to compute molecules can be deposited and maintained to avoid duplication of computational efforts. Till such a global archival norm is achieved, it is suggested that the chemical community should maintain a standard and consistent representation of chemical structure data in the electronic supplementary files in native format or standard data format to facilitate the re-usability among the scientific community.

## Conclusion

 Supplementary information of primary literature deposited with journals is a rich reservoir of peer reviewed molecular data which will be more valuable if available for further reuse. An application ChemEngine presented here selectively extracts the 3D structure from coordinate information present along with inadvertently introduced noisy data present in PDF files. This approach can obviate to some extent the loss of chemical data while at the same time conserve the memory and storage space required at the journal site. The methodology exemplified here will enable molecule mining in semantic context and ensure maximum reuse of the valuable data by interested readers thereby enhancing the citations of the authors. Further the application can be seamlessly integrated to enable a high throughput molecular computing automated workflow.

## Additional files

**Additional file 1.** Coordinate data of 29 molecules obtained as output from ChemEngine in the first case study pertaining to formation of epoxides from sulfur ylides and aldehydes.

**Additional file 2.** Recreated 3D geometry optimized structures of 29 molecules as visualized in the original program (Gauss View).

**Additional file 3.** A comparative table consisting of interatomic bond distances computed via ChemEngine and the commercial software package. This material is available free of charge via the Internet at http://pubs.acs.org.

**Additional file 4.** Sample input file containing co-ordinates of molecules separated by comma delimiter.

**Additional file 5.** Instruction for compilation of chemengine source code available online and operation manual.

## Abbreviations

PDF: printable document format
ORTEP: oak ridge thermal ellipsoid plot
SDF: structure data format
QMDFF: quantum mechanically derived force field
