# Supplementary material for: ChemEngine: harvesting 3D chemical structures of supplementary data from PDF files
Source: J Cheminform. 2016 Dec 29;8:73. doi: 10.1186/s13321-016-0175-x (PMC5195924; doi:10.1186/s13321-016-0175-x)

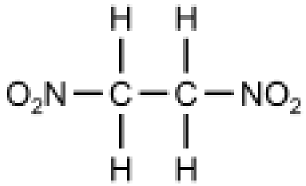

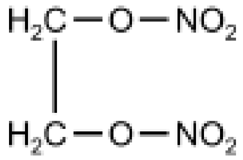

Chemical Name NITROGLYKOL
Molecular Formula (C2.0 H4.0 N2.0 O6.0)
Density(DICH)1.492(Ref.H)
Difference Enthalpy-Energy(DIFF)-3.55(Ref.528)
Enthalpy of Formation(ENTH)-58.02(Ref.C)
Enthalpy of Formation(ENTH)-58.24(Ref.9)
Enthalpy of Formation(ENTH)-54.39(Ref.102)
Enthalpy of Formation(ENTH)-67.7(Ref.549)
Enthalpy of Formation(ENTH)-59.6(Ref.549)
Melting Point(SCHM)-22.8(Ref.320)
Boiling Point(SIED)199.0(Ref.320)
Heat of Combustion(VBW)268.2(Ref.ME)
Classification Plasticizers (Energetic)(PE)
Oxygen Balance 0.0
Molecular Weight 152.064

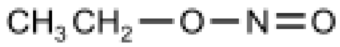

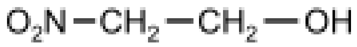

Chemical Name 2-NITROETHANOL
Molecular Formula (C2.0 H5.0 N O3.0)
Density(DICH)1.27(Ref.H)
Difference Enthalpy-Energy(DIFF)-2.66(Ref.528)
Enthalpy of Formation(ENTH)-83.6(Ref.STB)
Enthalpy of Formation(ENTH)-83.83(Ref.C)
Melting Point(SCHM)-80.0(Ref.H)
Boiling Point(SIED)194.0(Ref.H)
Heat of Combustion(VBW)275.06(Ref.C)
Classification N.A
Oxygen Balance -61.49
Molecular Weight 91.067

Opt=(Tight GDIIS) B3LYP/6-31G(d) SCRF
OCC[N+]([O-])=O
 0 1
C -0.4573800153984795 0.439422231460497 0.4932542685295179
C -0.03564186554515802 0.5792309120578124 -0.9883914402689875
O -0.6488664085471363 -0.9291872900921193 0.8307038635661743
N 0.16784703451227684 1.912565882535228 -1.3523565636751451
O 1.2062754539022207 2.2949584283739086 -1.7260866295903328
O -0.7833121522135728 2.7512786637647597 -1.304720254379945
H 0.37593089577962024 0.7698057028426903 1.1133669092239136
H -1.4387809258082567 0.8993611367556078 0.6090491580661288
H 0.9435964704430913 0.11516565457689179 -1.1060167697271093
H -0.8706729014086134 0.254678549764376 -1.6092689912197
H -0.6621911131937572 -1.0244228697663598 1.7858753856553706

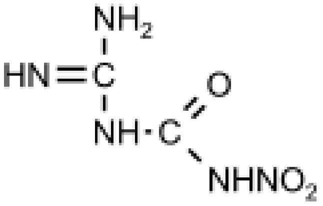

Chemical Name NITROGUANYLUREA

Molecular Formula (C2.0 H5.0 N5.0 O3.0)

Density(DICH)1.82(Ref.5)

Difference Enthalpy-Energy(DIFF)-3.85(Ref.528)

Enthalpy of Formation(ENTH)-73.6(Ref.C)

Enthalpy of Formation(ENTH)-74.9(Ref.SE)

Melting Point(SCHM)245.0(Ref.ME1)

Melting Point(SCHM)415.0(Ref.1467)

Heat of Combustion(VBW)286.6(Ref.C)

Classification N.A

Oxygen Balance -38.07

Molecular Weight 147.093

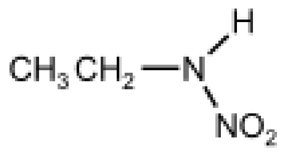

Supplement: Supplementary file 2 — Additional file 2. Recreated 3D geometry optimized structures of 29 molecules as visualized in the original program (Gauss View). [file 13321_2016_175_MOESM2_ESM.zip › hem_46_51.pdf]
