# Supplementary material for: ChemEngine: harvesting 3D chemical structures of supplementary data from PDF files
Source: J Cheminform. 2016 Dec 29;8:73. doi: 10.1186/s13321-016-0175-x (PMC5195924; doi:10.1186/s13321-016-0175-x)
Supplement: Supplementary file 2 — Additional file 2. Recreated 3D geometry optimized structures of 29 molecules as visualized in the original program (Gauss View). [file 13321_2016_175_MOESM2_ESM.zip › hem_1_6.pdf]

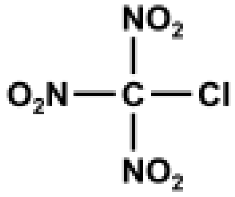

Chemical Name TRINITROCHLOROMETHANE
Molecular Formula ()
Density(DICH)1.677(Ref.H)
Difference Enthalpy-Energy(DIFF)-2.96(Ref.528)
Enthalpy of Formation(ENTH)-3.0(Ref.792)
Enthalpy of Formation(ENTH)-5.57(Ref.115)
Enthalpy of Formation(ENTH)-5.6(Ref.SE)
Enthalpy of Formation(ENTH)-6.54(Ref.C)
Melting Point(SCHM)4.5(Ref.H)
Boiling Point(SIED)133-135(Decomposition)(Ref.H)
Heat of Combustion(VBW)864.4(Ref.C)
Classification N.A
Oxygen Balance 38.82
Molecular Weight 185.48

Opt=(Tight GDIIS) B3LYP/6-31G(d) SCRF
[O-][N+](=O)C(Cl)([N+]([O-])=O)[N+]([O-])=O
0 1
C 0.7574948292859104 -1.2303612189448858 -1.5368095697960928
Cl 1.3230108966658705 -0.13001671483277163 -2.815008666195642
N 1.0880543671999154 -0.6913958074392853 -0.26685273107776686
O 1.2712453937346933 -1.3730137534053553 0.6655176585450424
O 1.1092467321962918 0.5605935056606512 -0.0374828073572394
N -0.6492359344927823 -1.3382907461075588 -1.680934270857215
O -1.1430917379319971 -1.6999527168676054 -2.678286240074922
O -1.4656622573268687 -1.071298889315576 -0.7422738355227265
N 1.4255214468678699 -2.4642580510104204 -1.744868844366563
O 2.5919919508550744 -2.543149564045913 -1.7113317811999083
O 0.8113771124719356 -3.5599506440741773 -1.9420469926311634

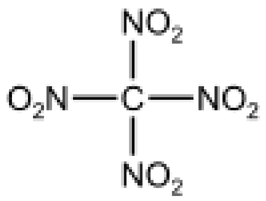

Chemical Name TETRANITROMETHANE
Molecular Formula (C N4.0 O8.0)
Density(DICH)1.638(Ref.H)
Difference Enthalpy-Energy(DIFF)-3.55(Ref.528)
Enthalpy of Formation(ENTH)9.2(Ref.525)
Enthalpy of Formation(ENTH)9.0(Ref.49)
Enthalpy of Formation(ENTH)8.8(Ref.STB)
Enthalpy of Formation(ENTH)13.0(Ref.29)
Enthalpy of Formation(ENTH)13.0(Ref.549)
Melting Point(SCHM)14.2(Ref.H)
Boiling Point(SIED)126.21(Ref.236)
Heat of Combustion(VBW)103.2(Ref.525)
Classification Monopropellants(M)
Oxygen Balance 48.97
Molecular Weight 196.033

Opt=(Tight GDIIS) B3LYP/6-31G(d) SCRF
[O-][N+](=O)C([N+]([O-])=O)([N+]([O-])=O)[N+]([O-])=O
 0 1
C 0.13325331120514533 -1.1955961972698277 1.6013759449536262
N 1.544020274260422 -0.9740545036684839 1.6087532087534415
O 2.055295004548298 -0.26693224379223646 2.3878208103148206
O 2.3563612948748784 -1.5480429117541588 0.8111564650103421
N -0.5256280158308867 0.05306440460050999 1.3808610320113828
O -0.40409506077110224 0.9534944915343815 2.117416225601679
O -1.3219380316745766 0.27178565473033334 0.4099271056286736
N -0.26101468725883076 -1.7227922846259265 2.8646986135672754
O -1.3277280348741334 -1.5559864208145782 3.315103164440488
O 0.49997036487846996 -2.4807000767935588 3.5474257643527882
N -0.2156049040521811 -2.128973719422448 0.5802904197698584

O 0.012086480913105036 -1.916980071961484 -0.5474763589635051
O -0.7880913028947133 -3.242243747231434 0.806063518042767

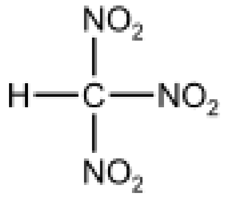

Chemical Name TRINITROMETHANE
Molecular Formula (C H N3.0 O6.0)
Density(DICH)1.479(Ref.H)
Difference Enthalpy-Energy(DIFF)-2.96(Ref.528)
Enthalpy of Formation(ENTH)-8.19(Ref.C)
Enthalpy of Formation(ENTH)-7.9(Ref.R)
Enthalpy of Formation(ENTH)-5.1(Ref.SE)
Enthalpy of Formation(ENTH)-9.2(Ref.STA)
Melting Point(SCHM)14.3(Ref.392)
Melting Point(SCHM)22.0(Ref.905)
Boiling Point(SIED)45-47 (22 Torr)(*)(Ref.392)
Heat of Combustion(VBW)120.4(Ref.C)
Density(DICH)1.61(Ref.958)
Melting Point(SCHM)26.0(Ref.958)
Classification N.A
Oxygen Balance 37.08
Molecular Weight 151.035

Opt=(Tight GDIIS) B3LYP/6-31G(d) SCRF
[O-][N+](=O)C([N+]([O-])=O)[N+]([O-])=O
0 1
C 2.2877304743743854 -1.111676075549894 -0.5890004664589866
N 1.4007092941690005 -2.135793724622468 -0.19938306289019045
O 0.46436685531037963 -2.3924896723231583 -0.8513425451597372
O 1.5537568533453 -2.8226096920489265 0.8598313296151255
N 1.5920767028825265 0.10960033758624282 -0.6848048153625709
O 1.0591311102810448 0.5913069883614412 0.23822025671785846
O 1.5124342542130254 0.7333447416982255 -1.7874450612010249
N 3.4489275560241524 -1.0216913289044747 0.2065192147963248
O 4.187461829433082 -1.923260958647023 0.2849927869220524

O 3.7731420623247662 0.021939465946795043 0.8561066434137654
H 2.669679942776489 -1.371889764249743 -1.576169874311848

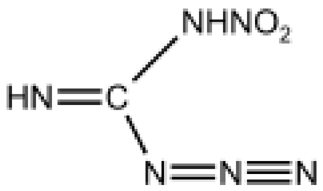

Chemical Name NITROGUANYL AZIDE
Molecular Formula (C H2.0 N6.0 O2.0)
Difference Enthalpy-Energy(DIFF)-2.96(Ref.528)
Enthalpy of Formation(ENTH)71.3(Ref.C)
Enthalpy of Formation(ENTH)71.3(Ref.SE)
Enthalpy of Formation(ENTH)71.3(Ref.STC)
Heat of Combustion(VBW)233.7(Ref.C)
Density(DICH)1.61(Ref.952)
Melting Point(SCHM)79.0(Ref.995)
Classification N.A
Oxygen Balance -12.3
Molecular Weight 130.066

Opt=(Tight GDIIS) B3LYP/6-31G(d) SCRF
[N-]=[N+]=[N-]
  -1 1
N 1.1610000000000003 0.0 0.0
N 0.0 0.0 0.0
N -1.1610000000000003 0.0 0.0

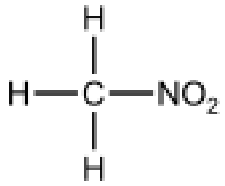

Chemical Name NITROMETHANE
Molecular Formula (C H3.0 N O2.0)
Density(DICH)1.139(Ref.236)
Difference Enthalpy-Energy(DIFF)-1.78(Ref.528)
Enthalpy of Formation(ENTH)-27.03(Ref.STA)
Enthalpy of Formation(ENTH)-27.03(Ref.SE)
Enthalpy of Formation(ENTH)-27.0(Ref.10)
Enthalpy of Formation(ENTH)-21.28(Ref.129)
Enthalpy of Formation(ENTH)-28.4(Ref.525)
Enthalpy of Formation(ENTH)-21.3(Ref.549)
Enthalpy of Formation(ENTH)-22.2(Ref.821)
Melting Point(SCHM)-29.2(Ref.236)
Boiling Point(SIED)101.15(Ref.236)
Heat of Combustion(VBW)169.5(Ref.C)
Classification Liquid fuels(LF)
Oxygen Balance -39.32
Molecular Weight 61.04

Opt=(Tight GDIIS) B3LYP/6-31G(d) SCRF
C[N+]([O-])=O
 0 1
C 1.3785102360397241 0.15999364795112594 0.028646332963262792
N -0.006752741274274932 0.03622436313371077 0.0013832731721754097
O -0.6713715721090698 0.779053935311926 -0.6073387322756082
O -0.5905341261593047 -0.8718199782290962 0.6696428587781447
H 1.720271296126639 0.19052902114605327 1.0632317420968516
H 1.8295648131106905 -0.6932324484385196 -0.47796495485316504
H 1.6711612547105152 1.0796677121325233 -0.47796495485316504

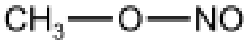

Chemical Name METHYL NITRITE
Molecular Formula (C H3.0 N O2.0)
Difference Enthalpy-Energy(DIFF)-1.18(Ref.528)
Enthalpy of Formation(ENTH)-15.3(Ref.ST)
Enthalpy of Formation(ENTH)-16.5(Ref.SE)
Enthalpy of Formation(ENTH)-15.79(Ref.C)
Enthalpy of Formation(ENTH)-16.05(Ref.49)
Melting Point(SCHM)-16.0(Ref.H)
Boiling Point(SIED)-12.0(Ref.H)
Heat of Combustion(VBW)179.7(Ref.C)
Classification N.A
Oxygen Balance -39.32
Molecular Weight 61.04

# Opt=(Tight GDIIS) B3LYP/6-31G(d) SCRF
CON=O
 0 1
O 0.06203252303095682 -0.06422594168626045 0.2851429289830533
N 1.2491376608273868 0.06424814669171194 -0.1606890406978569
O 1.8758494708951534 1.014467772888091 0.09030841540374779
C -0.4963972238355652 -1.3072465325060507 -0.15696087907308684
H -0.7679840509195947 -1.911777398125489 0.7084194002653332
H -1.3848628069390698 -1.1131414052624287 -0.7578369954384213
H 0.238777360459145 -1.8425653378817008 -0.7578369954384213
