# Supplementary material for: ChemEngine: harvesting 3D chemical structures of supplementary data from PDF files
Source: J Cheminform. 2016 Dec 29;8:73. doi: 10.1186/s13321-016-0175-x (PMC5195924; doi:10.1186/s13321-016-0175-x)
Supplement: Supplementary file 2 — Additional file 2. Recreated 3D geometry optimized structures of 29 molecules as visualized in the original program (Gauss View). [file 13321_2016_175_MOESM2_ESM.zip › hem_6_11.pdf]

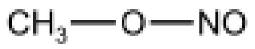

Chemical Name METHYL NITRITE
Molecular Formula (C H3.0 N O2.0)
Difference Enthalpy-Energy(DIFF)-1.18(Ref.528)
Enthalpy of Formation(ENTH)-15.3(Ref.ST)
Enthalpy of Formation(ENTH)-16.5(Ref.SE)
Enthalpy of Formation(ENTH)-15.79(Ref.C)
Enthalpy of Formation(ENTH)-16.05(Ref.49)
Melting Point(SCHM)-16.0(Ref.H)
Boiling Point(SIED)-12.0(Ref.H)
Heat of Combustion(VBW)179.7(Ref.C)
Classification N.A
Oxygen Balance -39.32
Molecular Weight 61.04

# Opt=(Tight GDIIS) B3LYP/6-31G(d) SCRF
CON=O
 0 1
O 0.06203252303095682 -0.06422594168626045 0.2851429289830533
N 1.2491376608273868 0.06424814669171194 -0.1606890406978569
O 1.8758494708951534 1.014467772888091 0.09030841540374779
C -0.4963972238355652 -1.3072465325060507 -0.15696087907308684
H -0.7679840509195947 -1.911777398125489 0.7084194002653332
H -1.3848628069390698 -1.1131414052624287 -0.7578369954384213
H 0.238777360459145 -1.8425653378817008 -0.7578369954384213

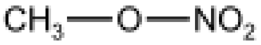

Chemical Name METHYL NITRATE
Molecular Formula (C H3.0 N O3.0)
Density(DICH)1.208(Ref.H)
Difference Enthalpy-Energy(DIFF)-2.07(Ref.528)
Enthalpy of Formation(ENTH)-37.26(Ref.C)
Enthalpy of Formation(ENTH)-37.19(Ref.10)
Enthalpy of Formation(ENTH)-36.9(Ref.ST)
Enthalpy of Formation(ENTH)-38.0(Ref.SE)
Enthalpy of Formation(ENTH)-42.0(Ref.549)
Melting Point(SCHM)-82.3(Ref.H)
Boiling Point(SIED)66.5(Ref.10)
Classification N.A
Oxygen Balance -10.38
Molecular Weight 77.04

Opt=(Tight GDIIS) B3LYP/6-31G(d) SCRF
CO[N+]([O-])=O
 0 1
C 1.4793787710809871 -0.02090964386484294 -0.03621654635256233
O 0.04435298206663986 0.042925759011950865 0.07434957545859959
N -0.46333403478451246 0.6373588932674065 1.1039378495500005
O -1.7253569680132466 0.6798735861080168 1.1775754370482452
O 0.1316327627356434 1.1412432404147117 1.9766910476009973
H 1.9200726937851207 -0.04051338500770802 0.9605304425191609
H 1.7623369914759093 -0.9243580906208702 -0.5764160924140715
H 1.8414382682610713 0.8538459625399655 -0.5764160924140715

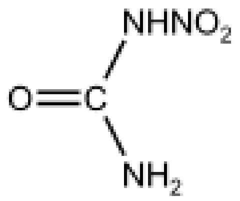

Chemical Name NITROUREA
Molecular Formula (C H3.0 N3.0 O3.0)
Density(DICH)1.69(Ref.952)
Difference Enthalpy-Energy(DIFF)-2.66(Ref.528)
Enthalpy of Formation(ENTH)-67.5(Ref.SE)
Enthalpy of Formation(ENTH)-67.2(Ref.STB)
Enthalpy of Formation(ENTH)-64.23(Ref.358)
Enthalpy of Formation(ENTH)-65.4(Ref.549)
Melting Point(SCHM)158-159(*)(Ref.H)
Heat of Combustion(VBW)129.1(Ref.L)
Density(DICH)1.5(Ref.CTI)
Classification N.A
Oxygen Balance -7.61
Molecular Weight 105.053

Opt=(Tight GDIIS) B3LYP/6-31G(d) SCRF
NC(=O)N[N+]([O-])=O
 0 1
N 0.28849405423550006 0.7573126712043068 -0.3777986562016576
C -1.019391834957971 0.5429251000573233 -0.6597200760246359
O -1.5771910356641992 -0.5615262474758199 -0.46346730771207867
N -1.7480934646280473 1.5487138213734242 -1.1693191920502901
N 1.058045291102095 -0.12004766391661034 0.11011117586310502
O 1.6895858931117071 -0.8045805881521352 -0.5921815054845956
O 1.171603536065982 -0.24195148376459577 1.3664000056315302
H 0.7289069318757456 1.650474266837957 -0.5463182731422077
H -1.3138842067854983 2.440238493395263 -1.361011861654986
H -2.7305248694247024 1.413871486444457 -1.361011861654986

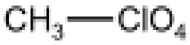

Chemical Name METHYLPERCHLORATE
Molecular Formula (C Cl H3.0 O4.0)
Difference Enthalpy-Energy(DIFF)-2.37(Ref.528)
Enthalpy of Formation(ENTH)-4.0(Ref.U2)
Classification N.A
Oxygen Balance 13.98
Molecular Weight 114.485

Opt=(Tight GDIIS) B3LYP/6-31G(d) SCRF
CO[Cl](=O)(=O)=O
 0 1
Cl 1.6565628638337406 -0.030904214231434537 0.09516269377434877
O 1.6324265227944257 -1.4264447266755558 0.764281475261712
O 2.4399023416855705 0.9455281870227782 1.0029704829312722
O 2.3720321124195154 -0.12457042269278355 -1.2742051113358286
O 0.1077257318808427 0.5345931192691386 -0.10563464064557695
C -0.6920418290296528 -0.31548813555416033 -0.9387093027932137
H -1.3037796971956377 -0.9657106752148655 -0.3133394685599664
H -1.3383367307082423 0.29637566629469153 -1.5680149424082832
H -0.04193292116763114 -0.9232979905804819 -1.5680149424082832

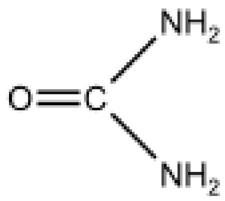

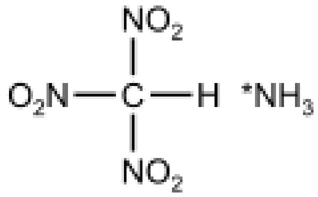

Chemical Name AMMONIUM TRINITROMETHANE
Molecular Formula (C H4.0 N4.0 O6.0)
Difference Enthalpy-Energy(DIFF)-4.14(Ref.528)
Enthalpy of Formation(ENTH)-47.3(Ref.R)
Heat of Combustion(VBW)186.3(Ref.R)
Density(DICH)1.75(Ref.952)
Classification N.A
Oxygen Balance 19.04
Molecular Weight 168.066

Opt=(Tight GDIIS) B3LYP/6-31G(d) SCRF
[NH4+].[O-][N+](=O)C([N+]([O-])=O)[N+]([O-])=O
 1 1
C -2.6497313550586967 -0.11654308369956978 -0.4223698944982483
N -3.536752535264082 -1.1406607327721436 -0.03275249092945212
O -4.473094974122702 -1.3973566804728341 -0.6847119731989988
O -3.383704976087782 -1.8274767001986023 1.0264619015758638
N -3.3453851265505556 1.104733329436567 -0.5181742434018326
O -3.8783307191520375 1.5864399802117655 0.4048508286785968
O -3.4250275752200565 1.7284777335485497 -1.6208144892402865
N -1.4885342734089297 -0.02655833705415045 0.3731497867570631
O -0.75 -0.9281279667966988 0.4516233588827907
O -1.1643197671083159 1.0170724577971193 1.0227372153745038
N 2.809488130197316 0.0 0.0

H -2.2677818866656593 -0.3767567723994188 -1.4095393023511094
H 2.809488130197316 1.01 0.0
H 2.809488130197316 -0.33666666666666667 0.0
H 1.9848265834603127 -0.33666666666666667 0.0
H 3.634149676934319 -0.33666666666666667 0.0
