# Supplementary material for: ChemEngine: harvesting 3D chemical structures of supplementary data from PDF files
Source: J Cheminform. 2016 Dec 29;8:73. doi: 10.1186/s13321-016-0175-x (PMC5195924; doi:10.1186/s13321-016-0175-x)
Supplement: Supplementary file 2 — Additional file 2. Recreated 3D geometry optimized structures of 29 molecules as visualized in the original program (Gauss View). [file 13321_2016_175_MOESM2_ESM.zip › hem_11_16.pdf]

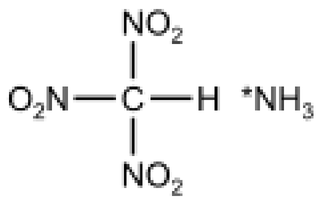

Chemical Name AMMONIUM TRINITROMETHANE
Molecular Formula (C H4.0 N4.0 O6.0)
Difference Enthalpy-Energy(DIFF)-4.14(Ref.528)
Enthalpy of Formation(ENTH)-47.3(Ref.R)
Heat of Combustion(VBW)186.3(Ref.R)
Density(DICH)1.75(Ref.952)
Classification N.A
Oxygen Balance 19.04
Molecular Weight 168.066

Opt=(Tight GDIIS) B3LYP/6-31G(d) SCRF
[NH4+].[O-][N+](=O)C([N+]([O-])=O)[N+]([O-])=O
1 1
C -2.6497313550586967 -0.11654308369956978 -0.4223698944982483
N -3.536752535264082 -1.1406607327721436 -0.03275249092945212
O -4.473094974122702 -1.3973566804728341 -0.6847119731989988
O -3.383704976087782 -1.8274767001986023 1.0264619015758638
N -3.3453851265505556 1.104733329436567 -0.5181742434018326
O -3.878307191520375 1.5864399802117655 0.4048508286785968
O -3.4250275752200565 1.7284777335485497 -1.6208144892402865
N -1.4885342734089297 -0.02655833705415045 0.3731497867570631
O -0.75 -0.9281279667966988 0.4516233588827907
O -1.1643197671083159 1.0170724577971193 1.0227372153745038
N 2.809488130197316 0.0 0.0
H -2.267781886656593 -0.3767567723994188 -1.4095393023511094
H 2.809488130197316 1.01 0.0
H 2.809488130197316 -0.33666666666666667 0.0
H 1.9848265834603127 -0.33666666666666667 0.0
H 3.634149676934319 -0.33666666666666667 0.0

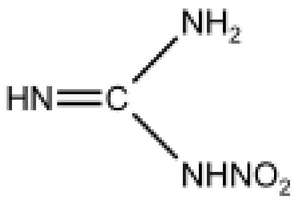

Chemical Name NITROGUANIDINE
Molecular Formula (C H4.0 N4.0 O2.0)
Classification null(classification)
Oxygen Balance oxybal
Molecular Weight molwt

Opt=(Tight GDIIS) B3LYP/6-31G(d) SCRF
NC(N)=N[N+]([O-])=O
0 1
N -0.8306918727332101 -2.1241050877430285 0.8845674891736154
C -1.4374774971159399 -1.0812292827931476 0.3494944867539006
N -2.7348905043995173 -1.148201131728109 0.16333954716097104
N -0.8525769288579812 0.008542409018413053 -0.002133091537822783
N 0.334635775700498 0.36013175511085693 0.02566865564508619
O 0.5648266472362895 1.4935766942185775 -0.11738445102135128
O 1.3191475328054647 -0.4332277828091569 0.16169651569485358
H -1.3545555197130272 -2.9630059739633907 1.0892921395499113
H 0.15749710646613357 -2.0832350630640226 1.0892921395499113
H -3.2792107531854735 -0.3004484711368469 0.09170929362218234
H -3.189029097043893 -2.047493767886637 0.09170929362218234

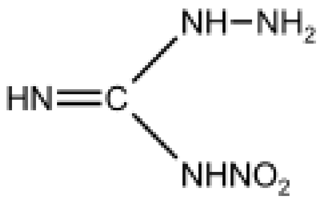

Chemical Name AMINONITROGUANIDINE
Molecular Formula (C H5.0 N5.0 O2.0)
Density(DICH)1.71(Ref.163)
Difference Enthalpy-Energy(DIFF)-3.55(Ref.528)
Enthalpy of Formation(ENTH)5.28(Ref.C)
Enthalpy of Formation(ENTH)5.3(Ref.STB)
Enthalpy of Formation(ENTH)5.98(Ref.163)
Melting Point(SCHM)190.0(Ref.225)
Heat of Combustion(VBW)270.1(Ref.C)
Classification N.A
Oxygen Balance -33.59
Molecular Weight 119.083

Opt=(Tight GDIIS) B3LYP/6-31G(d) SCRF
NNC(=N)N[N+]([O-])=O
0 1
N 0.2787956580955723 1.168155160468457 0.14663613609700238
C -0.9331162491329571 0.8333891554602457 -0.29886039751722093
N -1.5050856732853488 -0.3614912764028983 -0.21467138270098696
N -1.6220211165520333 1.7595470739878014 -0.8444407395149295
N 1.3235807834639122 0.4362617021587241 0.18017202043306782
O 2.2673759985433444 0.8474494398203273 0.7271949050357399
O 1.4336758117748034 -0.6912160439138859 -0.3982898237465708
N -1.1161854085596359 -1.3179840581252533 0.6500310571501208
H 0.42486560411009466 2.100422798775956 0.5066925503377058
H -2.274894463533597 -0.5524775367555066 -0.8399862432417025
H -2.046543150207879 2.3302709310172594 -0.1273944913843612
H -1.7806316854567874 -2.0060850639784964 0.9742657728442751
H -0.16008974811631338 -1.3471891470684103 0.9742657728442751

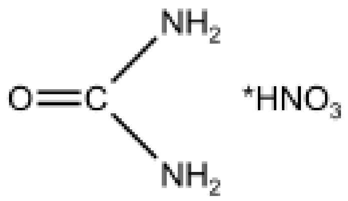

Chemical Name UREA NITRATE

Molecular Formula (C H5.0 N3.0 O4.0)

Density(DICH)1.69(Ref.H)

Difference Enthalpy-Energy(DIFF)-3.55(Ref.528)

Enthalpy of Formation(ENTH)-134.5(Ref.STB)

Enthalpy of Formation(ENTH)-138.8(Ref.SE)

Enthalpy of Formation(ENTH)-114.8(Ref.129)

Enthalpy of Formation(ENTH)-130.61(Ref.807)

Melting Point(SCHM)157.0(Ref.H)

Heat of Combustion(VBW)131.9(Ref.ME)

Melting Point(SCHM)140.0(Ref.1398)

Classification N.A

Oxygen Balance -6.5

Molecular Weight 123.068

```
# Opt=(Tight GDIIS) B3LYP/6-31G(d) SCRF
NC(N)=O.[O-][N+]([O-])=O
  -1  1
N -2.736012324024652 0.4376483712341588 -1.0392540497360556
C -2.0389153407275113 -0.001451753776141096 0.01839312592718023
O -2.5939842279838183 -0.09039371403762042 1.134982101305949
N -0.75 -0.34580290342039705 -0.1141211774970734
N 2.0172279731839953 -0.021650635094611015 0.0
O 1.434727973183995 -1.030570230503482 0.0
O 1.3847279731839959 1.073871500692704 0.0
O 3.2822279731839954 -0.021650635094611015 0.0
H -3.7289171770944267 0.603048602850264 -0.9562919129999011
H -2.268272443265148 0.603048602850264 -1.919005246000331
H -0.49026659628513075 -1.3205577956796488 -0.16403569453714067
H -0.03873502730605699 0.3695366520407428 -0.16403569453714067
```

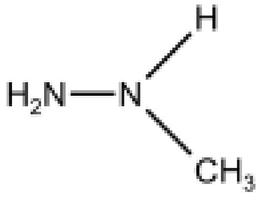

Chemical Name MONOMETHYLHYDRAZINE
Molecular Formula (C H6.0 N2.0)
Density(DICH)0.874(Ref.H)
Difference Enthalpy-Energy(DIFF)-2.37(Ref.528)
Enthalpy of Formation(ENTH)12.95(Ref.C)
Enthalpy of Formation(ENTH)12.7(Ref.STB)
Enthalpy of Formation(ENTH)13.05(Ref.10)
Enthalpy of Formation(ENTH)13.11(Ref.852)
Melting Point(SCHM)-52.4(Ref.H)
Boiling Point(SIED)87.5(Ref.H)
Heat of Combustion(VBW)311.95(Ref.C)
Classification Liquid fuels(LF)
Oxygen Balance -173.63
Molecular Weight 46.072

```
# Opt=(Tight GDIIS) B3LYP/6-31G(d) SCRF
CNN
  0  1
N 0.12156349471921418 -0.2395339445566017 -0.2798182152476386
N 1.0979087065975035 0.46525805809914855 0.43143395989795785
C 2.059597641008375 1.0811203522223478 -0.494941333713247
H -0.7429454851628561 0.21517751904589855 -0.5366483314173238
H 0.2809643204164866 -1.2032401592274908 -0.5366483314173238
H 1.1069342464544507 0.5200222115897996 1.4399077707243686
H 2.842412984890835 1.5824326126713364 0.07425648331147683
H 2.5045773312656583 0.3092492258662186 -1.1228808527405598
H 1.5446602751230674 1.8081908231251678 -1.1228808527405598
```

CH_3-NH_2 $*HNO_3$

Chemical Name METHYLAMINE NITRATE
Molecular Formula (C H6.0 N2.0 O3.0)
Density(DICH)1.422(Ref.EX)
Difference Enthalpy-Energy(DIFF)-3.26(Ref.528)
Enthalpy of Formation(ENTH)-84.3(Ref.STC)
Enthalpy of Formation(ENTH)-84.7(Ref.SE)
Enthalpy of Formation(ENTH)-58.0(Ref.853)
Melting Point(SCHM)110-111(*)(Ref.185)
Heat of Combustion(VBW)215.4(Ref.ME)
Classification N.A
Oxygen Balance -34.02
Molecular Weight 94.07

```
# Opt=(Tight GDIIS) B3LYP/6-31G(d) SCRF
CN.[O-][N+]([O-])=O
  -1  1
N -1.2707970570666518 -0.006807941745492287 -0.011791694444880103
C -2.734202942933348 0.006807941745492287 0.011791694444880103
N 1.99 -0.021650635094611015 0.0
O 1.4074999999999995 -1.030570230503482 0.0
O 1.3575000000000004 1.073871500692704 0.0
O 3.255 -0.021650635094611015 0.0
H -0.7740224042982983 -0.8861535709730004 -0.019928568866897797
H -0.7577465335297403 0.8631420289845155 -0.019928568866897797
H -3.0809157749128757 0.010033842072021782 1.0451743949522088
H -3.0974719480434367 0.9002076794661442 -0.4961182764820523
H -3.114032434727632 -0.8796778272301293 -0.4961182764820523
```
