# Supplementary material for: ChemEngine: harvesting 3D chemical structures of supplementary data from PDF files
Source: J Cheminform. 2016 Dec 29;8:73. doi: 10.1186/s13321-016-0175-x (PMC5195924; doi:10.1186/s13321-016-0175-x)
Supplement: Supplementary file 2 — Additional file 2. Recreated 3D geometry optimized structures of 29 molecules as visualized in the original program (Gauss View). [file 13321_2016_175_MOESM2_ESM.zip › hem_16_21.pdf]

CH_3-NH_2 $*HNO_3$

Chemical Name METHYLAMINE NITRATE
Molecular Formula (C H6.0 N2.0 O3.0)
Density(DICH)1.422(Ref.EX)
Difference Enthalpy-Energy(DIFF)-3.26(Ref.528)
Enthalpy of Formation(ENTH)-84.3(Ref.STC)
Enthalpy of Formation(ENTH)-84.7(Ref.SE)
Enthalpy of Formation(ENTH)-58.0(Ref.853)
Melting Point(SCHM)110-111(*)(Ref.185)
Heat of Combustion(VBW)215.4(Ref.ME)
Classification N.A
Oxygen Balance -34.02
Molecular Weight 94.07

Opt=(Tight GDIIS) B3LYP/6-31G(d) SCRF
CN.[O-][N+]([O-])=O
-1 1
N -1.2707970570666518 -0.006807941745492287 -0.011791694444880103
C -2.734202942933348 0.006807941745492287 0.011791694444880103
N 1.99 -0.021650635094611015 0.0
O 1.4074999999999995 -1.030570230503482 0.0
O 1.3575000000000004 1.073871500692704 0.0
O 3.255 -0.021650635094611015 0.0
H -0.7740224042982983 -0.8861535709730004 -0.019928568866897797
H -0.7577465335297403 0.8631420289845155 -0.019928568866897797
H -3.0809157749128757 0.010033842072021782 1.0451743949522088
H -3.0974719480434367 0.9002076794661442 -0.4961182764820523
H -3.114032434727632 -0.8796778272301293 -0.4961182764820523

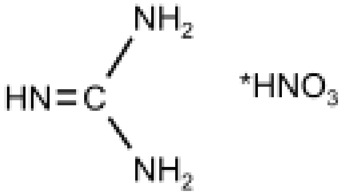

Chemical Name GUANIDINE NITRATE
Molecular Formula (C H6.0 N4.0 O3.0)
Density(DICH)1.436(Ref.30)
Difference Enthalpy-Energy(DIFF)-3.85(Ref.528)
Enthalpy of Formation(ENTH)-92.5(Ref.SE)
Enthalpy of Formation(ENTH)-92.0(Ref.10)
Enthalpy of Formation(ENTH)-93.0(Ref.SA)
Enthalpy of Formation(ENTH)-96.9(Ref.30)
Enthalpy of Formation(ENTH)-92.47(Ref.525)
Melting Point(SCHM)216.0(Ref.10)
Boiling Point(SIED)240.0(Ref.1400)
Boiling Point(SIED)270.0(Ref.1401)
Heat of Combustion(VBW)206.53(Ref.525)
Classification Energetic fillers(E)
Oxygen Balance -26.21
Molecular Weight 122.084

Opt=(Tight GDIIS) B3LYP/6-31G(d) SCRF
NC(N)=N.O[N+]([O-])=O
0 1
N -2.643925992767297 0.42578098033713185 -1.0620280068307513
C -2.0143348496214557 -0.0013663954190596406 0.00715883936757189
N -0.75 -0.35332364651849657 -0.05974879188693494
N -2.6450183128949227 -0.07109093839957586 1.1146179593501144
O 3.2547435323995737 -0.1652852651594869 -0.04965189502987266
N 2.0011310526059516 -0.00639457720280831 0.020245645521190014
O 1.3946509060140313 0.6702242692512679 -0.8609243454020454
O 1.402753664264119 -0.4985444268889726 0.8903305949107281
H -3.639933602446112 0.5901623128605957 -1.0296610641313966
H -2.1324960109953257 0.5901623128605957 -1.917316350760757

H -0.4986994478422916 -1.3312228899431227 -0.08546069953251237
H -0.029559054901753118 0.3540685660702272 -0.08546069953251237
H -2.7856323380810646 -1.0402975217499506 1.3615314962638372
H 3.6213108854657334 -0.2117463046353617 0.8363897222076535

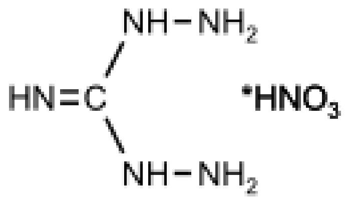

Chemical Name DIAMINOGUANIDINE NITRATE
Molecular Formula (C H8.0 N6.0 O3.0)
Density(DICH)1.61(Ref.807)
Difference Enthalpy-Energy(DIFF)-5.03(Ref.528)
Enthalpy of Formation(ENTH)-37.6(Ref.SE)
Enthalpy of Formation(ENTH)-37.6(Ref.STB)
Enthalpy of Formation(ENTH)-37.57(Ref.C)
Enthalpy of Formation(ENTH)-36.46(Ref.358)
Enthalpy of Formation(ENTH)-47.08(Ref.807)
Melting Point(SCHM)142-144(*)(Ref.852)
Heat of Combustion(VBW)329.74(Ref.C)
Classification N.A
Oxygen Balance -31.55
Molecular Weight 152.113

Opt=(Tight GDIIS) B3LYP/6-31G(d) SCRF
[O-][N+]([O-])=O.NNC(=N)NN
-1 1
N -2.534395830401213 1.0783126809350474 0.5116645749128836
C -2.274127220328906 -0.2264232226476784 0.5405182814367195
N -2.4033225136385354 -1.0998081788237823 -0.45537236777691864
N -1.8513105918724406 -0.6929854291038984 1.6507840764710624
N -3.088740901288967 1.7330155442032118 -0.5276124588194149
N -2.746196207689209 -0.7921113945628997 -1.7199821062243321
N 2.4705155442032116 -0.021650635094611015 0.0
O 1.8880155442032114 -1.030570230503482 0.0
O 1.8380155442032122 1.073871500692704 0.0
O 3.7355155442032117 -0.021650635094611015 0.0
H -2.2942700049019233 1.6090920570691478 1.3367178106492086
H -2.227858518772038 -2.069664372628318 -0.23471120253829184

H -1.8636629370828761 -1.7023888865286387 1.6183482982811923
H -4.068237757419648 1.9783587205443032 -0.5054170842342398
H -2.5247142931474778 1.9783587205443032 -1.3287246301982243
H -3.7190524241398664 -0.6766603499234378 -1.9655871517852253
H -2.0306395678501743 -0.6766603499234378 -2.423366603831386

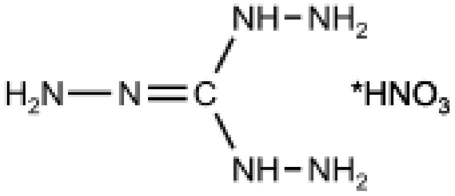

Chemical Name TRIAMINOGUANIDINE NITRATE
Molecular Formula (C H9.0 N7.0 O3.0)
Density(DICH)1.59(Ref.807)
Difference Enthalpy-Energy(DIFF)-5.62(Ref.528)
Enthalpy of Formation(ENTH)-11.5(Ref.74)
Enthalpy of Formation(ENTH)-12.0(Ref.69)
Enthalpy of Formation(ENTH)-11.2(Ref.10)
Enthalpy of Formation(ENTH)-3.23(Ref.163)
Melting Point(SCHM)215-217(*)(Ref.395)
Heat of Combustion(VBW)389.47(Ref.525)
Boiling Point(SIED)190.0(Ref.1400)
Classification Energetic fillers(E)
Oxygen Balance -33.51
Molecular Weight 167.128

Opt=(Tight GDIIS) B3LYP/6-31G(d) SCRF
NNC(NN)=NN
 0 1
N -2.373270325795261 -0.42675596069802024 1.0441567497666573
N -1.87545727100724 0.7543400954541957 0.6189213917720533
C -0.6264806158347127 0.9427539897535403 0.20929042342447024
N -0.3481587839974836 2.203099341845846 -0.11340174104626645
N 0.4635031783935953 2.5926463308934773 -1.1208368579565928
N 0.18722927228529987 -0.054718639149309745 0.17137713622148176
N 1.412435429462414 -0.07065153172974747 -0.13332753827505994
H -3.3654483853265424 -0.5287131853797695 1.2031769450876824
H -1.7534146882708153 -1.2081595479109442 1.2031769450876824
H -2.484857946175856 1.5596788783123157 0.6062619317268366
H -0.786290938481175 2.9184491553418583 0.4491059118249633
H 0.0857517623298007 5 2.738247269899462 -2.0461499045642655

H 1.4480042149255459 2.738247269899462 -0.9486216564554177
H 1.8910946201017724 -0.95163575164188 -0.25519707295163757
H 1.9138419664313622 0.797587664115799 -0.25519707295163757

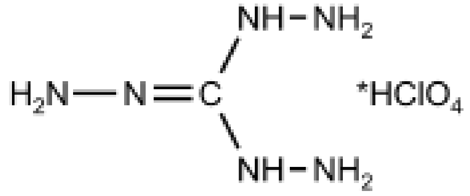

Chemical Name TRIAMINOGUANIDINE PERCHLORATE
Molecular Formula (C Cl H9.0 N6.0 O4.0)
Density(DICH)1.67(Ref.69)
Difference Enthalpy-Energy(DIFF)-5.92(Ref.528)
Enthalpy of Formation(ENTH)8.68(Ref.69)
Enthalpy of Formation(ENTH)-23.8(Ref.10)
Melting Point(SCHM)115.0(Ref.69)
Melting Point(SCHM)135.0(Ref.1399)
Classification N.A
Oxygen Balance -15.64
Molecular Weight 204.573

Opt=(Tight GDIIS) B3LYP/6-31G(d) SCRF
[O-][Cl](=O)(=O)=O
 -1 1
Cl -1.4878397821220753E-8 1.0222398683124685E-8 -1.7705713065178128E-8
O -0.5156667381633594 -0.7292627981561786 1.2631201789130688
O -0.515666647706583 1.458525594030854 5.159436652195907E-9
O -0.5156666477065831 -0.7292627672208237 -1.26312023370243
O 1.647 -4.61344182689764E-9 7.99071539213092E-9

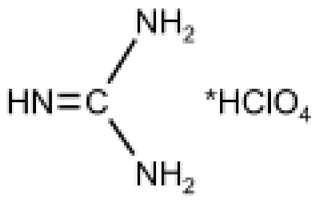

Chemical Name GUANIDINE PERCHLORATE
Molecular Formula (C Cl H6.0 N3.0 O4.0)
Density(DICH)1.82(Ref.952)
Difference Enthalpy-Energy(DIFF)-4.14(Ref.528)
Enthalpy of Formation(ENTH)-74.35(Ref.525)
Enthalpy of Formation(ENTH)-74.1(Ref.10)
Enthalpy of Formation(ENTH)-74.8(Ref.SE)
Melting Point(SCHM)240.0(Ref.188)
Boiling Point(SIED)275-325(Decomposition)(Ref.188)
Density(DICH)1.75(Ref.SA)
Melting Point(SCHM)246.0(Ref.965)
Melting Point(SCHM)178.0(Ref.1399)
Classification N.A
Oxygen Balance -5.01
Molecular Weight 159.529

Opt=(Tight GDIIS) B3LYP/6-31G(d) SCRF
NC(N)=N.[O-][Cl](=O)(=O)=O
 -1 1
Cl -2.397000014878398 3.369836982493116E-9 -5.836728597141691E-9
O -2.9126667381633595 -0.7292628050087403 1.2631201907820533
O -2.9126666477065832 1.4585255871782923 1.7028421120232344E-8
O -2.9126666477065832 -0.7292627740733855 -1.2631202218334456
O -0.75 -1.146600352752921E-8 1.9859699860167358E-8
N 1.7463938057446067 0.42578098033713185 -1.0620280068307513
C 2.375984948890448 -0.0013663954190596406 0.00715883936757189
N 3.6403197985119036 -0.35332364651849657 -0.05974879188693494
N 1.7453014856169808 -0.07109093839957586 1.1146179593501144
H 0.7503861960657914 0.5901623128605957 -1.0296610641313966
H 2.257823787516578 0.5901623128605957 -1.917316350760757

H 3.891620350669612 -1.3312228899431227 -0.08546069953251237
H 4.36076074361015 0.3540685660702272 -0.08546069953251237
H 1.6046874604308388 -1.0402975217499506 1.3615314962638372
