# Supplementary material for: ChemEngine: harvesting 3D chemical structures of supplementary data from PDF files
Source: J Cheminform. 2016 Dec 29;8:73. doi: 10.1186/s13321-016-0175-x (PMC5195924; doi:10.1186/s13321-016-0175-x)
Supplement: Supplementary file 2 — Additional file 2. Recreated 3D geometry optimized structures of 29 molecules as visualized in the original program (Gauss View). [file 13321_2016_175_MOESM2_ESM.zip › hem_21_26.pdf]

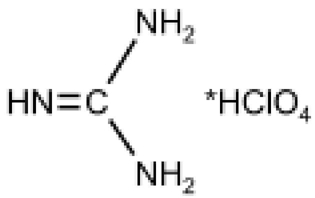

Chemical Name GUANIDINE PERCHLORATE
Molecular Formula (C Cl H6.0 N3.0 O4.0)
Density(DICH)1.82(Ref.952)
Difference Enthalpy-Energy(DIFF)-4.14(Ref.528)
Enthalpy of Formation(ENTH)-74.35(Ref.525)
Enthalpy of Formation(ENTH)-74.1(Ref.10)
Enthalpy of Formation(ENTH)-74.8(Ref.SE)
Melting Point(SCHM)240.0(Ref.188)
Boiling Point(SIED)275-325(Decomposition)(Ref.188)
Density(DICH)1.75(Ref.SA)
Melting Point(SCHM)246.0(Ref.965)
Melting Point(SCHM)178.0(Ref.1399)
Classification N.A
Oxygen Balance -5.01
Molecular Weight 159.529

Opt=(Tight GDIIS) B3LYP/6-31G(d) SCRF
NC(N)=N.[O-][Cl](=O)(=O)=O
 -1 1
Cl -2.397000014878398 3.369836982493116E-9 -5.836728597141691E-9
O -2.9126667381633595 -0.7292628050087403 1.2631201907820533
O -2.9126666477065832 1.4585255871782923 1.7028421120232344E-8
O -2.9126666477065832 -0.7292627740733855 -1.2631202218334456
O -0.75 -1.146600352752921E-8 1.9859699860167358E-8
N 1.7463938057446067 0.42578098033713185 -1.0620280068307513
C 2.375984948890448 -0.0013663954190596406 0.00715883936757189
N 3.6403197985119036 -0.35332364651849657 -0.05974879188693494
N 1.7453014856169808 -0.07109093839957586 1.1146179593501144
H 0.7503861960657914 0.5901623128605957 -1.0296610641313966
H 2.257823787516578 0.5901623128605957 -1.917316350760757
H 3.891620350669612 -1.3312228899431227 -0.08546069953251237

H 4.36076074361015 0.3540685660702272 -0.08546069953251237
H 1.6046874604308388 -1.0402975217499506 1.3615314962638372

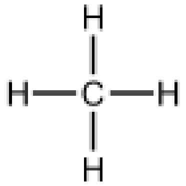

Chemical Name METHANE
Molecular Formula (C H4.0)
Density(DICH)0.72(Ref.RP)
Difference Enthalpy-Energy(DIFF)-0.59(Ref.528)
Enthalpy of Formation(ENTH)-17.8(Ref.743)
Enthalpy of Formation(ENTH)-17.89(Ref.ST)
Enthalpy of Formation(ENTH)-17.9(Ref.JA)
Enthalpy of Formation(ENTH)-17.89(Ref.C)
Melting Point(SCHM)-182.0(Ref.H)
Boiling Point(SIED)-164.0(Ref.H)
Heat of Combustion(VBW)212.9(Ref.743)
Classification N.A
Oxygen Balance -398.92
Molecular Weight 16.043

Opt=(Tight GDIIS) B3LYP/6-31G(d) SCRF
C
0 1
C 9.158592157371484E-9 1.2952205027506736E-8 -5.494395096234187E-16
H 9.158592157371484E-9 1.0900000129522052 -5.494395096234187E-16
H 9.158592157371484E-9 -0.3633333203811283 -5.494395096234187E-16
H -0.8899812640526293 -0.3633333203811283 -5.494395096234187E-16
H 0.8899812823698136 -0.3633333203811283 -5.494395096234187E-16

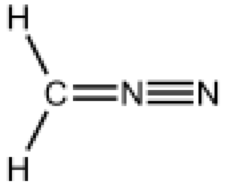

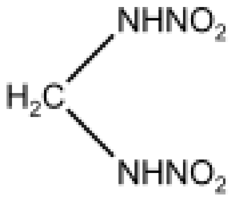

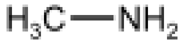

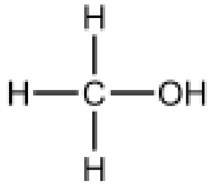

Chemical Name METHANOL
Molecular Formula (C H4.0 O)
Density(DICH)0.791(Ref.H)
Difference Enthalpy-Energy(DIFF)-1.48(Ref.528)
Enthalpy of Formation(ENTH)-57.01(Ref.C)
Enthalpy of Formation(ENTH)-57.02(Ref.ST)
Melting Point(SCHM)-97.7(Ref.135)
Boiling Point(SIED)64.5(Ref.135)
Heat of Combustion(VBW)173.45(Ref.C)
Classification Solvents(S)
Oxygen Balance -149.8
Molecular Weight 32.042

Opt=(Tight GDIIS) B3LYP/6-31G(d) SCRF
CO

0 1

C 1.4100156155263794 0.0012955465678606932 0.0022439686215908763

O -0.011101468291854764 -0.009706255201567902 -0.016811727124996786

H 1.759527597355242 0.004001348632944514 1.0346847917166706

H 1.7870840327822355 -0.8857932614330207 -0.5066694379887473

H 1.7733045852534481 0.8941159479067334 -0.5066694379887473

H -0.3431977008458324 -0.01227723050018041 0.8839132488856989
