# Supplementary material for: ChemEngine: harvesting 3D chemical structures of supplementary data from PDF files
Source: J Cheminform. 2016 Dec 29;8:73. doi: 10.1186/s13321-016-0175-x (PMC5195924; doi:10.1186/s13321-016-0175-x)
Supplement: Supplementary file 2 — Additional file 2. Recreated 3D geometry optimized structures of 29 molecules as visualized in the original program (Gauss View). [file 13321_2016_175_MOESM2_ESM.zip › hem_26_31.pdf]

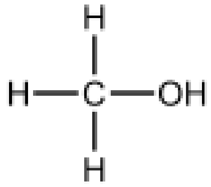

Chemical Name METHANOL
Molecular Formula (C H4.0 O)
Density(DICH)0.791(Ref.H)
Difference Enthalpy-Energy(DIFF)-1.48(Ref.528)
Enthalpy of Formation(ENTH)-57.01(Ref.C)
Enthalpy of Formation(ENTH)-57.02(Ref.ST)
Melting Point(SCHM)-97.7(Ref.135)
Boiling Point(SIED)64.5(Ref.135)
Heat of Combustion(VBW)173.45(Ref.C)
Classification Solvents(S)
Oxygen Balance -149.8
Molecular Weight 32.042

```
# Opt=(Tight GDIIS) B3LYP/6-31G(d) SCRF
CO
  0  1
C 1.4100156155263794 0.0012955465678606932 0.0022439686215908763
O -0.011101468291854764 -0.009706255201567902 -0.016811727124996786
H 1.759527597355242 0.004001348632944514 1.0346847917166706
H 1.7870840327822355 -0.8857932614330207 -0.5066694379887473
H 1.7733045852534481 0.8941159479067334 -0.5066694379887473
H -0.3431977008458324 -0.01227723050018041 0.8839132488856989
```

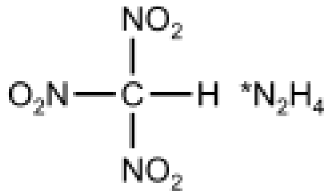

Chemical Name HYDRAZINIUM NITROFORMATE
Molecular Formula (C H5.0 N5.0 O6.0)
Density(DICH)1.91(Ref.322)
Density(DICH)1.87(Ref.1003)
Difference Enthalpy-Energy(DIFF)-4.74(Ref.528)
Enthalpy of Formation(ENTH)-18.37(Ref.525)
Enthalpy of Formation(ENTH)-17.2(Ref.278)
Enthalpy of Formation(ENTH)-17.2(Ref.SA)
Enthalpy of Formation(ENTH)-16.52(Ref.531)
Enthalpy of Formation(ENTH)-11.0(Ref.1410)
Melting Point(SCHM)118-124(*)(Ref.407)
Heat of Combustion(VBW)248.33(Ref.531)
Classification Energetic fillers(E)
Oxygen Balance 13.11
Molecular Weight 183.081

Opt=(Tight GDIIS) B3LYP/6-31G(d) SCRF
N[NH3+].[O-]C(=O)[N+]([O-])=O
 0 1
C -1.601895567655369 0.10065459354127182 -0.026154089846206596
O -0.757431071278917 -0.4499609436001194 0.7183206248979939
O -1.1357479419065029 0.9705299505503732 -0.9534153027740089
N -2.899866817416933 -0.1485139633585758 0.0784721876756076
O -3.326117586827541 -0.8943340233483403 0.8698193367094058
O -3.7355294925426312 0.42162438621539 -0.6870427566627912
N 2.9397059397780745 -0.0069880910273658016 0.012103728042353793
N 1.5458235527645567 0.006988091027365801 -0.012103728042353793
H 3.259771653020407 -0.010197326411519466 0.9700429954361308
H 3.27630326962743 -0.8350660857408471 -0.458097369584591
H 3.29283987370194 0.8141741051932537 -0.458097369584591

H 1.0496949229159422 0.8866923000770479 -0.020872264112297485
H 1.0321552055976178 -0.8625910841453008 -0.020872264112297485

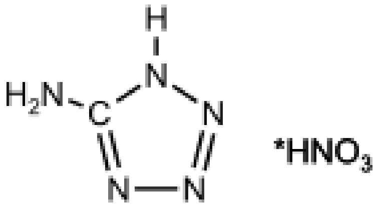

Chemical Name 5-AMINOTETRAZOLE NITRATE
Molecular Formula (C H4.0 N6.0 O3.0)
Density(DICH)1.8(Ref.419)
Difference Enthalpy-Energy(DIFF)-3.85(Ref.528)
Enthalpy of Formation(ENTH)-6.59(Ref.C)
Enthalpy of Formation(ENTH)-6.6(Ref.SE)
Melting Point(SCHM)160.0(Ref.419)
Heat of Combustion(VBW)224.09(Ref.C)
Classification N.A
Oxygen Balance -10.8
Molecular Weight 148.081

Opt=(Tight GDIIS) B3LYP/6-31G(d) SCRF
[O-][N+]([O-])=O.NC1=NN=NN1
 -1 1
N -3.809536308612443 -0.2598248293222898 0.029701343761524272
N -3.205930780476553 -1.397913188461409 0.07487417431009444
N -1.9297558252823916 -1.172033817682502 0.04197743799790275
N -1.6970423300524544 0.10120439485591226 -0.025240762469417567
C -2.8901760104349563 0.7033244011369575 -0.03477788928791129
N -3.1190169819811873 2.025243039473331 -0.08653430431219258
N 2.7627430394733308 -0.021650635094611015 0.0
O 2.1802430394733303 -1.030570230503482 0.0
O 2.1302430394733314 1.073871500692704 0.0
O 4.0277430394733305 -0.021650635094611015 0.0
H -4.812858732645117 -0.1445757885278992 0.04242030713013037
H -2.343226918372703 2.671672295816032 -0.10600206613952942
H -4.066960450276157 2.3732720428140555 -0.10600206613952942

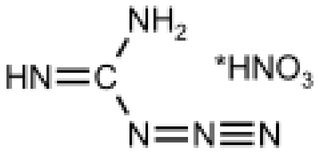

Chemical Name GUANYL AZIDE NITRATE
Molecular Formula (C H4.0 N6.0 O3.0)
Difference Enthalpy-Energy(DIFF)-3.85(Ref.528)
Enthalpy of Formation(ENTH)3.8(Ref.SE)
Enthalpy of Formation(ENTH)3.79(Ref.C)
Enthalpy of Formation(ENTH)4.69(Ref.230)
Heat of Combustion(VBW)234.47(Ref.C)
Density(DICH)1.5(Ref.999)
Classification Oxidizers(O)
Oxygen Balance -10.8
Molecular Weight 148.081

```
# Opt=(Tight GDIIS) B3LYP/6-31G(d) SCRF
[N-]=[N+]=[N-]
  -1  1
N 1.1610000000000003 0.0 0.0
N 0.0 0.0 0.0
N -1.1610000000000003 0.0 0.0

# Opt=(Tight GDIIS) B3LYP/6-31G(d) SCRF
[O-][N+]([O-])=O
  -1  1
N 0.0 0.0 0.0
O -0.5825000000000004 -1.008919595408871 0.0
O -0.6324999999999995 1.095522135787315 0.0
O 1.265 0.0 0.0
```

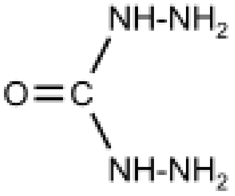

Chemical Name CARBOHYDRAZIDE
Molecular Formula (C H6.0 N4.0 O)
Density(DICH)1.616(Ref.B033)
Difference Enthalpy-Energy(DIFF)-3.26(Ref.528)
Enthalpy of Formation(ENTH)-27.96(Ref.10)
Melting Point(SCHM)154.0(Ref.H)
Heat of Combustion(VBW)271.6(Ref.10)
Classification N.A
Oxygen Balance -71.04
Molecular Weight 90.085

Opt=(Tight GDIIS) B3LYP/6-31G(d) SCRF
NNC(=O)NN
0 1
N 2.4104871152348206 -2.7797385930760097 2.1127329020709458
N 1.5129192095619348 -2.073105156587446 1.3970993991297638
C 1.7199971394965579 -0.7903393294801361 1.025599155731012
O 2.7616989568030004 -0.17429496782337447 1.3577904901649178
N 0.7596012318959519 -0.19868812691526438 0.2821493302311701
N 0.8382271316380804 1.0852548883124988 -0.1230010847380559
H 2.205680516474625 -3.7317247525039114 2.3808310358988285
H 3.287806332551053 -2.357205302593899 2.3808310358988285
H 0.6497252951754293 -2.523307345771049 1.1281862997456065
H -0.05166169063591719 -0.739226039802902 0.01800687764899317
H 1.7407188200254033 1.5125641578058466 -0.27470982079785056
H -0.005381557099107814 1.6194915771408323 -0.27470982079785056

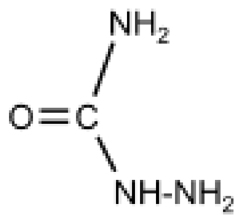
NH2
O=C
NH-NH2
