# Supplementary material for: ChemEngine: harvesting 3D chemical structures of supplementary data from PDF files
Source: J Cheminform. 2016 Dec 29;8:73. doi: 10.1186/s13321-016-0175-x (PMC5195924; doi:10.1186/s13321-016-0175-x)
Supplement: Supplementary file 2 — Additional file 2. Recreated 3D geometry optimized structures of 29 molecules as visualized in the original program (Gauss View). [file 13321_2016_175_MOESM2_ESM.zip › hem_31_36.pdf]

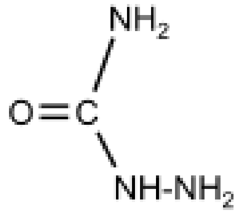

Chemical Name SEMICARBAZIDE
Molecular Formula (C H5.0 N3.0 O)
Difference Enthalpy-Energy(DIFF)-2.66(Ref.622)
Enthalpy of Formation(ENTH)-53.94(Ref.622)
Heat of Combustion(VBW)210.9(Ref.622)
Classification N.A
Oxygen Balance -74.59
Molecular Weight 75.07

Opt=(Tight GDIIS) B3LYP/6-31G(d) SCRF
NNC(N)=O
0 1
N 0.9082578204929963 0.8064627381149918 -0.23990302431139834
N 0.9595809915587422 -0.4657497839374837 0.1958995452741486
C 1.7449964961306885 -0.8458401651119087 1.225571887340624
N 1.7970926896803605 -2.14180605122445 1.5690077894737946
O 2.4327663998069013 -0.02629447012519441 1.8768745264620723
H 1.762973504009623 1.319119779642068 -0.40343851930686203
H 0.015023960303131467 1.2486045851357206 -0.40343851930686203
H 0.388817756052937 -1.1593825823647692 -0.26583941771346603
H 0.9427211178987844 -2.6647209679581336 1.6982718880815828
H 2.6906807059761384 -2.594455197853152 1.6982718880815828

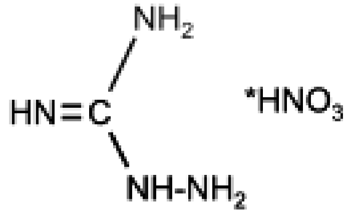

Chemical Name AMINOGUANIDINE NITRATE
Molecular Formula (C H7.0 N5.0 O3.0)
Difference Enthalpy-Energy(DIFF)-4.44(Ref.528)
Enthalpy of Formation(ENTH)-66.62(Ref.525)
Melting Point(SCHM)144-146(*)(Ref.852)
Heat of Combustion(VBW)226.53(Ref.525)
Classification N.A
Oxygen Balance -29.18
Molecular Weight 137.098

Opt=(Tight GDIIS) B3LYP/6-31G(d) SCRF
[O-][N+]([O-])=O.NN=C(N)N
-1 1
N -1.3688358089659718 0.9430196417114178 0.3052488719041837
C -2.5570618980426416 0.4007670351192749 0.11393415260171791
N -3.6153943774406185 1.1471500524131857 0.3206044676459452
N -2.7468615644264016 -0.8123901051003115 -0.25705941395494897
N -1.8545794718422013 -1.678546624143567 -0.4827280781968977
N 2.4160466241435667 -0.021650635094611015 0.0
O 1.8335466241435665 -1.030570230503482 0.0
O 1.7835466241435673 1.073871500692704 0.0
O 3.681046624143567 -0.021650635094611015 0.0
H -0.5511247358654745 0.35472392723553514 0.37843871585909356
H -1.2774053130355765 1.9462064195129287 0.37843871585909356
H -3.5188182610678385 2.1493673702896605 0.40018776674089784
H -4.527042462245902 0.7197576472677244 0.40018776674089784
H -2.1072880862199517 -2.65225477294842 -0.5728990112646173
H -0.888808553327511 -1.3970226717327847 -0.5728990112646173

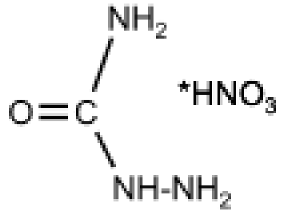

Chemical Name SEMICARBAZIDE NITRATE
Molecular Formula (C H6.0 N4.0 O4.0)
Density(DICH)1.75(Ref.952)
Difference Enthalpy-Energy(DIFF)-4.14(Ref.528)
Enthalpy of Formation(ENTH)-114.24(Ref.952)
Melting Point(SCHM)115-119(*)(Ref.97)
Heat of Combustion(VBW)235.2(Ref.ICT)
Classification N.A
Oxygen Balance -11.59
Molecular Weight 138.083

Opt=(Tight GDIIS) B3LYP/6-31G(d) SCRF
[O-][N+]([O-])=O.NNC(N)=O
-1 1
N -3.0174415638085827 1.3411082845718008 -1.1653931691592467
N -2.9661183927428367 0.06889576251932528 -0.7295905995736998
C -2.1807028881708903 -0.3111946186550998 0.30008174249277575
N -2.1286066946212183 -1.607160504767641 0.6435176446259463
O -1.4929329844946775 0.5083510763316146 0.951384381614224
N 2.3446605047676408 -0.021650635094611015 0.0
O 1.7621605047676405 -1.030570230503482 0.0
O 1.7121605047676414 1.073871500692704 0.0
O 3.609660504767641 -0.021650635094611015 0.0
H -2.1627258802919562 1.853765326098877 -1.3289286641547104
H -3.910675423998448 1.7832501315925295 -1.3289286641547104
H -3.5368816282486417 -0.6247370359079601 -1.1913295625613145
H -2.982978266402794 -2.1300754215013247 0.7727817432337345
H -1.2350186783254402 -2.059809651396343 0.7727817432337345

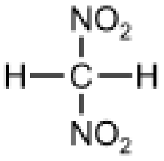

Chemical Name DINITROMETHANE
Molecular Formula (C H2.0 N2.0 O4.0)
Density(DICH)1.524(Ref.12)
Difference Enthalpy-Energy(DIFF)-2.37(Ref.528)
Enthalpy of Formation(ENTH)-25.2(Ref.12)
Melting Point(SCHM)< -15(*)(Ref.H)
Boiling Point(SIED)100.0(Ref.H)
Heat of Combustion(VBW)137.3(Ref.12)
Classification N.A
Oxygen Balance 15.09
Molecular Weight 106.038

```
# Opt=(Tight GDIIS) B3LYP/6-31G(d) SCRF
[O-][N+](=O)C[N+]([O-])=O
  0  1
C -0.05441640615070148 -0.08293554793053222 -0.026032607590242764
N -0.8273834270756054 1.0321366033626527 0.30905043002273114
O -1.1607442062158233 1.804523008997037 -0.5007282065574744
O -1.162802173577957 1.2546593809841025 1.51257170654671
N 1.3115926194215468 0.19612860409206273 0.03833591642193679
O 1.8144625306238318 0.5128577153135324 1.0444644074221776
O 2.054784468358084 0.111128416715328 -0.9889041326189849
H -0.24937604545866396 -0.8323104653480631 0.7411231602863848
H -0.27202628468504453 -0.3034382074099 -1.071080222738373
```

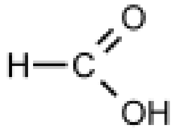

Chemical Name FORMIC ACID
Molecular Formula (C H2.0 O2.0)
Density(DICH)1.22(Ref.H)
Difference Enthalpy-Energy(DIFF)-1.18(Ref.528)
Enthalpy of Formation(ENTH)-101.52(Ref.ST)
Enthalpy of Formation(ENTH)-101.6(Ref.C)
Melting Point(SCHM)8.0(Ref.RP)
Boiling Point(SIED)101.0(Ref.RP)
Classification N.A
Oxygen Balance -34.76
Molecular Weight 46.026

# Opt=(Tight GDIIS) B3LYP/6-31G(d) SCRF
OC=O
 0 1
C 1.354595592940829 -0.2422940535901912 -0.24577953652121048
O 1.8007013361584494 0.2233570085786851 -1.317017888950878
O 0.14218317077377288 0.1436063847129854 0.2054819965196633
H 1.9958373943746022 -0.9548683181456514 0.2730141311225021
H 0.14309001722019551 0.1433177432946447 1.1654815248089505
