# Supplementary material for: ChemEngine: harvesting 3D chemical structures of supplementary data from PDF files
Source: J Cheminform. 2016 Dec 29;8:73. doi: 10.1186/s13321-016-0175-x (PMC5195924; doi:10.1186/s13321-016-0175-x)

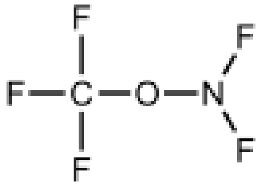

Chemical Name TRIFLUOROMETHOXYDIFLUOROAMINE
Molecular Formula (C F5.0 N O)
Difference Enthalpy-Energy(DIFF)-1.48(Ref.528)
Enthalpy of Formation(ENTH)-189.1(Ref.39)
Classification N.A
Oxygen Balance 17.52
Molecular Weight 137.009

Opt=(Tight GDIIS) B3LYP/6-31G(d) SCRF
FN(F)OC(F)(F)F
 0 1
N 1.4019614542353322 -0.12231928581237403 0.016748849554652438
F 1.788113127358512 -1.3149781305410908 0.3839511527847748
F 2.2705741492912574 0.7055961273556137 0.5280421318778641
O 0.21430939901376292 0.15422521918347318 0.6255284917840827
C -0.9162511484990264 -0.15074010869476687 -0.207702643983676
F -1.0192674307652787 -1.4950889688575177 -0.47384349511000884
F -2.0434172153651082 0.22917376991038774 0.4733297065857297
F -0.8849191801064943 0.552926038947007 -1.3891535642716308

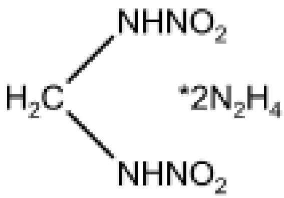

Chemical Name DIHYDRAZINE-METHYLENEDINITRAMINE SALT
Molecular Formula (C H12.0 N8.0 O4.0)
Density(DICH)1.4(Ref.41)
Difference Enthalpy-Energy(DIFF)-7.1(Ref.528)
Enthalpy of Formation(ENTH)-10.8(Ref.41)
Classification N.A
Oxygen Balance -31.97
Molecular Weight 200.157

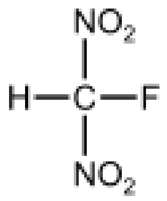

Chemical Name FLUORODINITROMETHANE
Molecular Formula (C F H N2.0 O4.0)
Density(DICH)1.595(Ref.239)
Difference Enthalpy-Energy(DIFF)-2.37(Ref.528)
Enthalpy of Formation(ENTH)-66.5(Ref.49)
Classification N.A
Oxygen Balance 25.8
Molecular Weight 124.028

Opt=(Tight GDIIS) B3LYP/6-31G(d) SCRF
[O-][N+](=O)C(F)[N+]([O-])=O
 0 1
C 0.2929918188520238 0.5041318214051043 0.4341780373426154
N 0.96430704744923 -0.14740381888590978 -0.6056323430744609
O 1.4901242009141928 0.4477825140176748 -1.4617968109073545
O 1.0215424346290674 -1.415714223208706 -0.6554348730571862
N -0.7738834891296269 1.2721095151784936 -0.04721939982275802
O -1.8407888333339975 0.8113288299662913 -0.16645553050955358
O -0.6157925571771365 2.4781216081877497 -0.4145472002658019
F 1.1851905279462265 1.276075665529946 1.1480383236717262
H -0.12370551798555157 -0.24125529806510015 1.1115711075761276

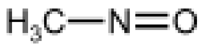

Chemical Name NITROSOMETHANE
Molecular Formula (C H3.0 N O)
Difference Enthalpy-Energy(DIFF)-0.89(Ref.528)
Enthalpy of Formation(ENTH)16.7(Ref.49)
Classification N.A
Oxygen Balance -88.8
Molecular Weight 45.041

# Opt=(Tight GDIIS) B3LYP/6-31G(d) SCRF
CN=O
0 1
C 1.4020600574603024 -0.1690862189243901 -0.19283329291264384
N 0.03194130740113851 -0.02577939770140926 -0.07185556818357665
O -0.47578062392485576 1.0185566013184348 0.05232303825244582
H 1.8514503592810696 -0.21608995173192172 0.7991037039433045
H 1.6247478223270784 -1.087214352297816 -0.7364792163654629
H 1.8099119386598457 0.6830909650667827 -0.7364792163654629

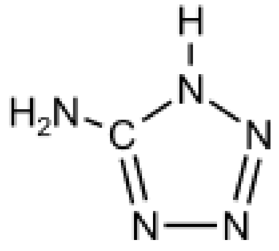

Supplement: Supplementary file 2 — Additional file 2. Recreated 3D geometry optimized structures of 29 molecules as visualized in the original program (Gauss View). [file 13321_2016_175_MOESM2_ESM.zip › hem_36_41.pdf]
