# Supplementary material for: ChemEngine: harvesting 3D chemical structures of supplementary data from PDF files
Source: J Cheminform. 2016 Dec 29;8:73. doi: 10.1186/s13321-016-0175-x (PMC5195924; doi:10.1186/s13321-016-0175-x)
Supplement: Supplementary file 2 — Additional file 2. Recreated 3D geometry optimized structures of 29 molecules as visualized in the original program (Gauss View). [file 13321_2016_175_MOESM2_ESM.zip › hem_41_46.pdf]

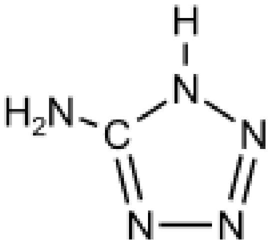

Chemical Name 5-AMINO-1H-TETRAZOLE
Molecular Formula (C H3.0 N5.0)
Density(DICH)1.65(Ref.358)
Difference Enthalpy-Energy(DIFF)-2.37(Ref.528)
Enthalpy of Formation(ENTH)49.66(Ref.C)
Enthalpy of Formation(ENTH)50.0(Ref.266)
Enthalpy of Formation(ENTH)49.67(Ref.765)
Melting Point(SCHM)209-210(*)(Ref.90)
Heat of Combustion(VBW)246.18(Ref.C)
Classification N.A
Oxygen Balance -65.83
Molecular Weight 85.068

# Opt=(Tight GDIIS) B3LYP/6-31G(d) SCRF
NC1=NN=NN1
0 1
N -0.5287844426017433 1.0372358402210073 0.005016352181154501
N 0.07482108553414707 -0.10085251891811205 0.05018918272972467
N 1.3509960407283084 0.12502685186079512 0.01729244641753298
N 1.5837095359582456 1.3982650643992094 -0.04992575404978734
C 0.39057585557574365 2.0003850706802546 -0.05946288086828106
N 0.16173488402951267 3.322303709016628 -0.11121929589256235
H -1.5321068666344173 1.1524848810153978 0.017735315549760593
H 0.9375249476379971 3.968732965359329 -0.1306870577198992
H -0.786208584265457 3.6703327123573524 -0.1306870577198992

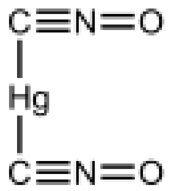

Chemical Name MERCURY FULMINATE

Molecular Formula (C2.0 Hg N2.0 O2.0)

Density(DICH)4.42(Ref.EX)

Difference Enthalpy-Energy(DIFF)-1.18(Ref.528)

Enthalpy of Formation(ENTH)64.05(Ref.SE)

Enthalpy of Formation(ENTH)64.1(Ref.ME)

Enthalpy of Formation(ENTH)65.4(Ref.192)

Heat of Combustion(VBW)267.0(Ref.27)

Melting Point(SCHM)160.0(Ref.325)

Classification Primary explosives(PX)

Oxygen Balance -16.86

Molecular Weight 284.624

```
# Opt=(Tight GDIIS) B3LYP/6-31G(d) SCRF
[O-][N+]#C[Hg]C#[N+][O-]
  0  1
O 1.3511773480469085 1.2153533998908714 0.0
N 0.34607157256850324 0.5486266611094179 0.0
C -0.6084133041213635 -0.08949487834326095 0.0
Hg -2.348552407170017 -1.260712921025598 0.0
C -4.032919113515015 -2.510713202596308 0.0
N -4.952358820136058 -3.1983876024840905 0.0
O -5.916338506917695 -3.9234583012346174 0.0
```

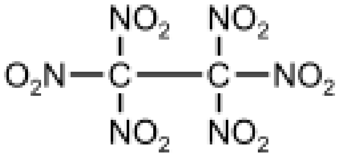

Chemical Name HEXANITROETHANE
Molecular Formula (C2.0 N6.0 O12.0)
Density(DICH)1.85(Ref.807)
Density(DICH)1.998(Ref.585)
Density(DICH)1.05(Ref.EX)
Difference Enthalpy-Energy(DIFF)-5.33(Ref.528)
Enthalpy of Formation(ENTH)19.0(Ref.1431)
Enthalpy of Formation(ENTH)28.6(Ref.SE)
Enthalpy of Formation(ENTH)20.0(Ref.49)
Enthalpy of Formation(ENTH)20.4(Ref.91)
Melting Point(SCHM)150.0(Ref.148)
Heat of Combustion(VBW)207.1(Ref.1431)
Enthalpy of Formation(ENTH)28.6(Ref.STB)
Classification Oxidizers(O)
Oxygen Balance 42.66
Molecular Weight 300.055

Opt=(Tight GDIIS) B3LYP/6-31G(d) SCRF
[O-][N+](=O)C([N+]([O-])=O)([N+]([O-])=O)C([N+]([O-])=O)([N+]([O-])=O)[N+]([O-])=O
0 1
C -0.8112732997790182 -1.4019709571947454 -2.9050681744280955
C 0.43790572825390245 -1.5535316419201617 -1.8828550887817783
N 1.2621289960794806 -0.38510263280261026 -1.7813210674380573
O 1.22831898397134 0.5257341596017665 -2.5111086717827833
O 2.1152539533857886 -0.2503846041780644 -0.844543663783541
N 1.3505253037029614 -2.5994975989190547 -2.2310369541215103
O 1.2880205085440326 -3.2502987967803674 -3.1983264500191093
O 2.317699551812532 -2.9237169583469997 -1.4668566272050687
N -0.09240885756137104 -1.838368697974265 -0.5851392097501931
O -0.4681645570824622 -0.9992363054472304 0.13885086513408915
O -0.24033504447815615 -3.021486732707384 -0.1382609021554773

N -1.7671639541215938 -0.48610427912028453 -2.3543917410439326
O -1.4998746781846488 0.6265373808992053 -2.114878688560369
O -2.9785293388724376 -0.7878458067833822 -2.105624084023195
N -0.43429597278606025 -0.8895667512947577 -4.188273377305485
O -1.1970025256992625 -0.3598046677452976 -4.9004422469355715
O 0.7136169947264984 -1.0696034031565553 -4.702851884781366
N -1.4217971833596994 -2.6842521714359338 -3.1200067493916435
O -1.9549316013638143 -3.284801224097195 -2.2711426007072624
O -1.3973734401243265 -3.299568643834688 -4.2358626334588685

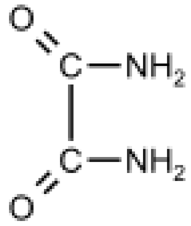

Chemical Name OXALIC ACID DIAMIDE
Molecular Formula (C2.0 H4.0 N2.0 O2.0)
Density(DICH)1.667(Ref.RP)
Difference Enthalpy-Energy(DIFF)-2.37(Ref.528)
Enthalpy of Formation(ENTH)-120.6(Ref.925)
Enthalpy of Formation(ENTH)-121.2(Ref.STC)
Enthalpy of Formation(ENTH)-123.0(Ref.SE)
Enthalpy of Formation(ENTH)-119.3(Ref.99)
Enthalpy of Formation(ENTH)-123.2(Ref.C)
Enthalpy of Formation(ENTH)-109.2(Ref.926)
Melting Point(SCHM)350.0(Ref.RP)
Heat of Combustion(VBW)201.5(Ref.C)
Classification Coolants(CO)
Oxygen Balance -72.67
Molecular Weight 88.066

```
# Opt=(Tight GDIIS) B3LYP/6-31G(d) SCRF
NC(=O)C(N)=O
  0  1
C -1.2083069900046146 1.0326240373823994 -0.01133552471416529
O -2.436094387931727 1.2439448207626456 0.11774857398349195
C -0.6959269687754001 -0.27886239386611134 0.004193874691208729
O 0.5392968387182189 -0.48388723948102264 0.015173864461411772
N -0.4050241847506071 2.102513769203523 -0.17842347802194677
N -1.5069350885677437 -1.3536093520570582 -0.003023801157563231
H 0.5953243390000247 1.9780703294561697 -0.24100671326732648
H -0.8036293060049817 3.028417271722013 -0.24100671326732648
H -2.5093197360020856 -1.2298446957584928 -0.005730910174251859
H -1.1129145294350655 -2.2835773464211346 -0.005730910174251859
```

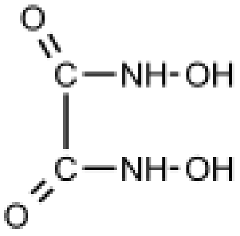

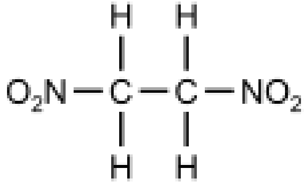

Chemical Name 1,2-DINITROETHANE
Molecular Formula (C2.0 H4.0 N2.0 O4.0)
Density(DICH)1.46(Ref.DL)
Difference Enthalpy-Energy(DIFF)-2.96(Ref.528)
Enthalpy of Formation(ENTH)-42.7(Ref.1431)
Enthalpy of Formation(ENTH)-43.9(Ref.STB)
Enthalpy of Formation(ENTH)-42.0(Ref.C)
Melting Point(SCHM)39.0(Ref.60)
Heat of Combustion(VBW)282.0(Ref.1431)
Classification N.A
Oxygen Balance -26.65
Molecular Weight 120.065

Opt=(Tight GDIIS) B3LYP/6-31G(d) SCRF
[O-][N+](=O)CC[N+]([O-])=O
0 1
C -0.2180829684141754 -0.04029404107813525 -0.3443331785638602
C -0.4611234089892891 -1.3962010364656474 0.37171283077600764
N -0.7773045173068811 1.0238883602239126 0.36890492072472775
O -0.16723563230608282 1.6050251220543945 1.177931455884263
O -1.9804831053800314 1.3821872984578065 0.17001087191473124
N 0.09463199294251856 -2.4716387991745425 -0.32687286292709705
O 0.9261628304505688 -3.1462177067857837 0.14131722477167857

O -0.31092306799929154 -2.767648660124924 -1.4939480351528083
H 0.8530686613473761 0.16138997845615877 -0.336734341569787
H -0.7639690004892747 -0.05097699701500506 -1.2877282784227448
H 0.08219684709612596 -1.3816475400106736 1.3165362508224276
H -1.5325773980184971 -1.5961902286868468 0.36219062577346345
