# Supplementary material for: ChemEngine: harvesting 3D chemical structures of supplementary data from PDF files
Source: J Cheminform. 2016 Dec 29;8:73. doi: 10.1186/s13321-016-0175-x (PMC5195924; doi:10.1186/s13321-016-0175-x)
Supplement: Supplementary file 5 — Additional file 5. Instruction for compilation of chemengine source code available online and operation manual. [file 13321_2016_175_MOESM5_ESM.docx]

**Compilation and Operation manual (ChemEngine)**

========================

BUILD OUTPUT DESCRIPTION

========================

When you build an Java application project that has a main class, the IDE

automatically copies all of the JAR

files on the projects classpath to your projects dist/lib folder. The IDE

also adds each of the JAR files to the Class-Path element in the application

JAR files manifest file (MANIFEST.MF).

To run the project from the command line, go to the dist folder and

type the following:

java -jar "ChemEngine.jar"

To distribute this project, zip up the dist folder (including the lib folder)

and distribute the ZIP file.

Notes:

To compile and execute the program please follow the steps:

1) Download ChemEngine_source.zip

unzip to ChemEngine folder

2) Download lib.zip folder

unzip all the jar files to ChemEngine\lib folder

open the ChemEngine project in Netbeans

Select all the libraries (jars) from lib folder

ChemEngine>Project properties>Libraries>Add Jar/folders > OK

ChemEngine>Source Packages>chemengine>ChemEngineGUI (Run)

To Transform PDF to Molecules in GUI

Select the pdf file and click

* If two JAR files on the project classpath have the same name, only the first

JAR file is copied to the lib folder.

* Only JAR files are copied to the lib folder.

If the classpath contains other types of files or folders, these files (folders)

are not copied.

* If a library on the projects classpath also has a Class-Path element

specified in the manifest,the content of the Class-Path element has to be on

the projects runtime path.

* To set a main class in a standard Java project, right-click the project node

in the Projects window and choose Properties. Then click Run and enter the

class name in the Main Class field. Alternatively, you can manually type the

class name in the manifest Main-Class element.

Figure-1: Netbean interface for compiling ChemEngine


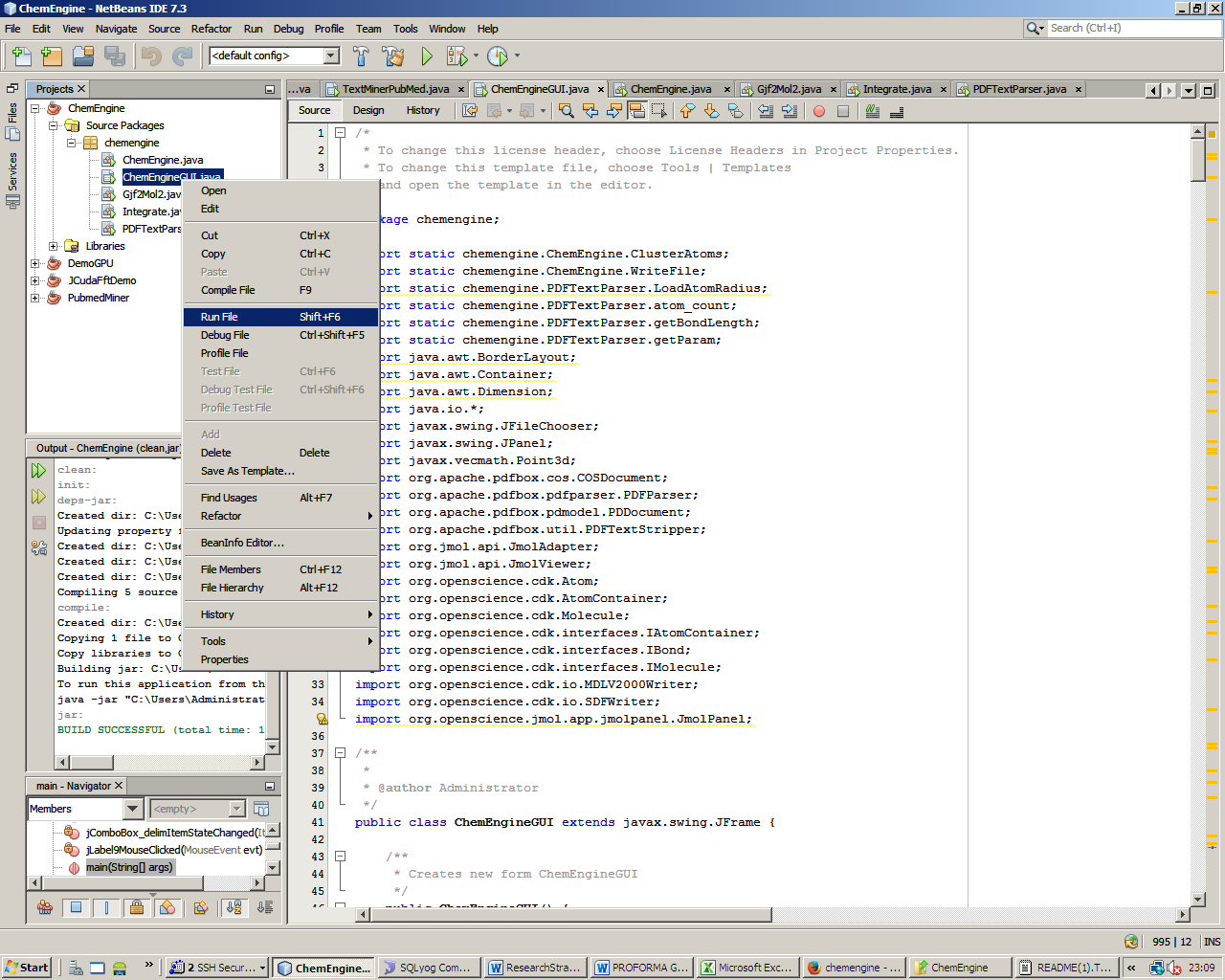


Figure-2: User Interface of ChemEngine (Browse and select PDF file) with default regular expression


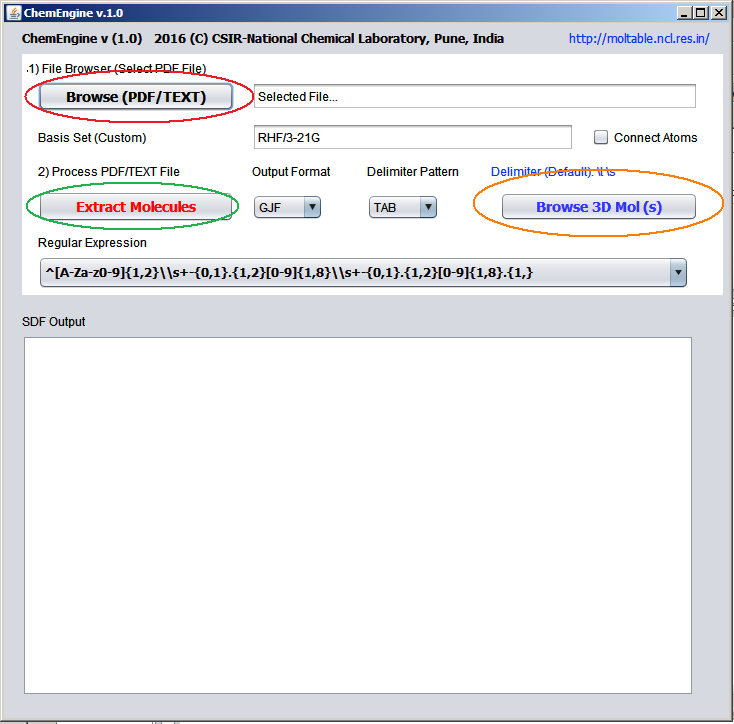


Figure-3; Select the Pdf file


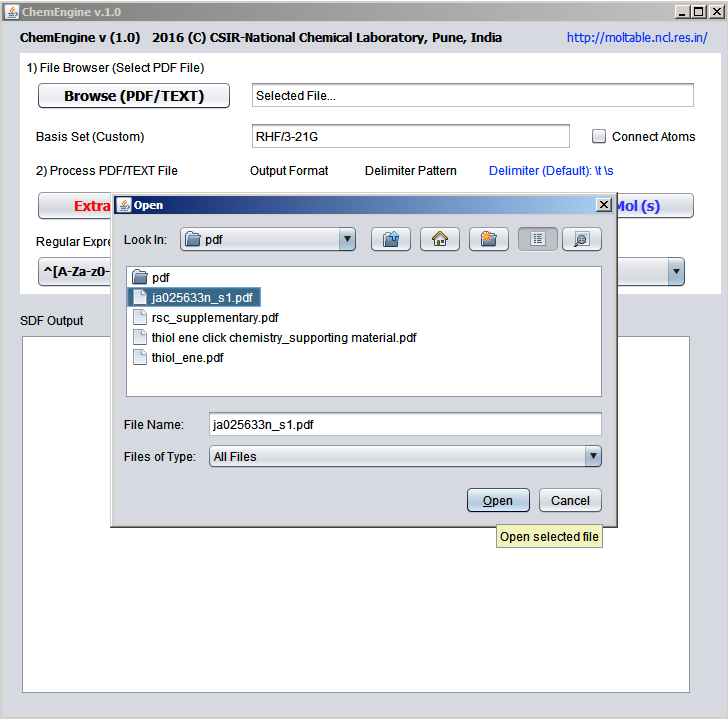


Figure-4: Select and click the button Extract Molecules to process the data from the selected pdf file


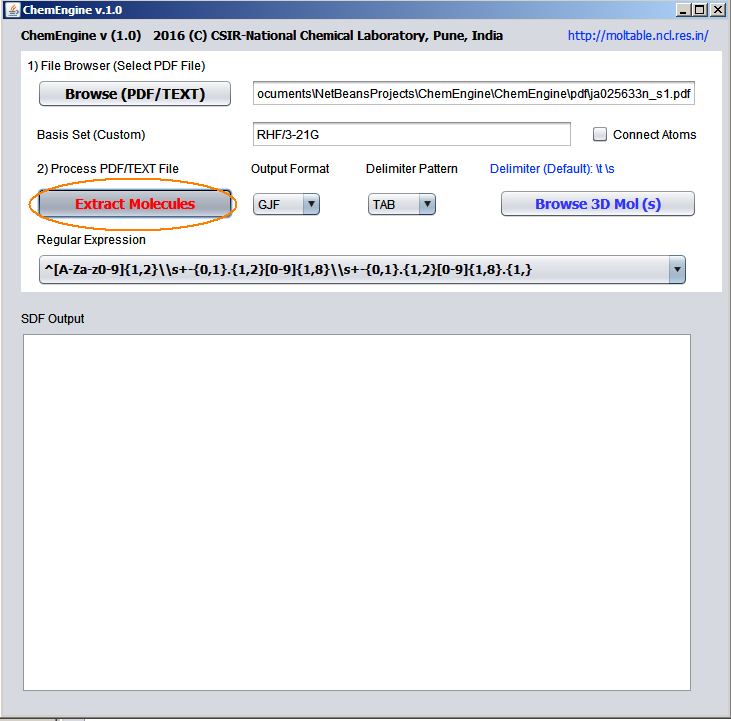


Figure-5: The generated SDF files are displayed in the Text Area


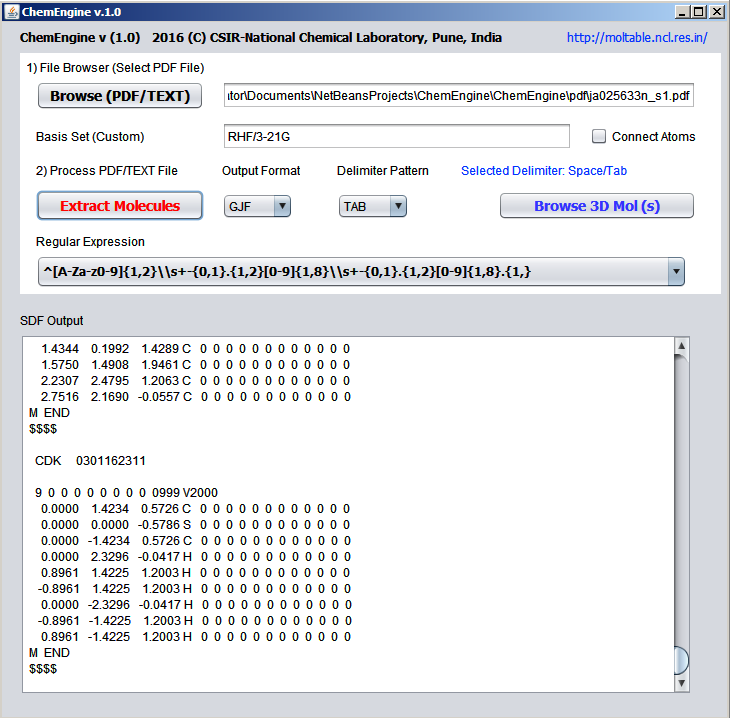


Figure-6: On click of Browse 3D mol(s) button, the output 3D molecules are displayed in the Jmol interface


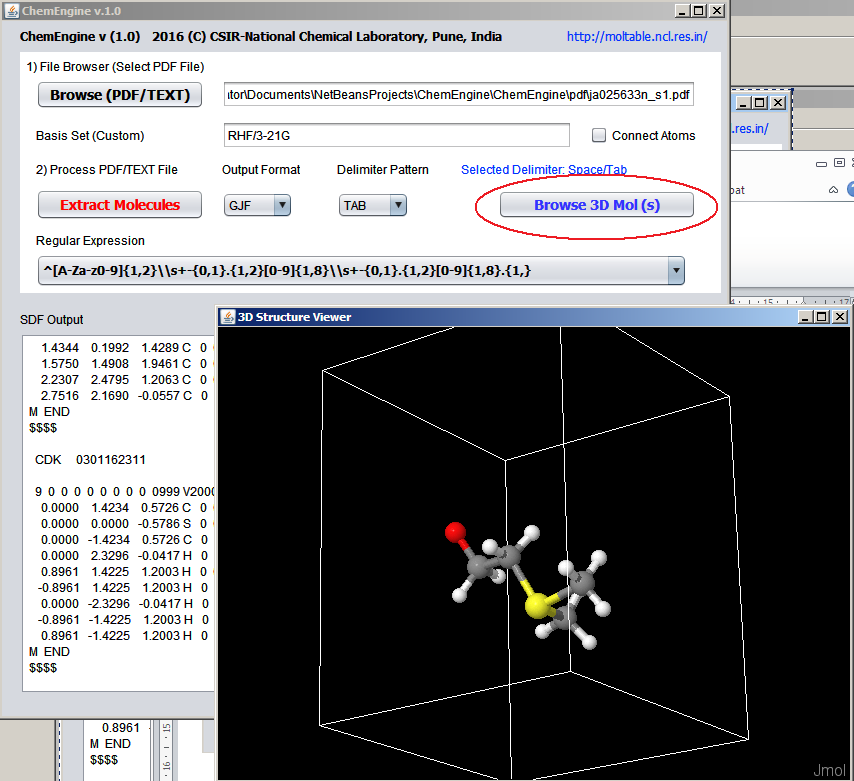


Contact:

Dr M Karthikeyan

[karthincl@gmail.com](mailto:karthincl@gmail.com)

<http://moltable.ncl.res.in/>
